# Coping With Chronic Illness: A Systematic Review of Adaptive Strategies Across Cancer, COPD, Diabetes and Heart Disease

**DOI:** 10.1002/puh2.70129

**Published:** 2025-10-25

**Authors:** Andrew Kweku Conduah, Mary Naana Essiaw, Sebastian Hadjor Ofoe

**Affiliations:** ^1^ Institute of Work, Employment & Society (IWES), Department of Business Administration University of Professional Studies, Accra (UPSA) Accra Ghana; ^2^ Regional Institute for Population Studies (RIPS) University of Ghana Accra Ghana; ^3^ Joshua Alabi Library, Department of Electronic Information Resources University of Professional Studies, Accra (UPSA) Accra Ghana

**Keywords:** cancer, chronic disease, chronic obstructive pulmonary disease (COPD), coping strategies, coronary heart disease, diabetes mellitus, emotional adaptation, psychological resilience, social determinants of health

## Abstract

**Background:**

Chronic diseases such as cancer, chronic obstructive pulmonary disease (COPD), diabetes and coronary heart disease profoundly affect individuals’ quality of life, requiring sustained adaptive coping mechanisms to manage physical, emotional and social challenges. This review contributes to the public health literature by systematically synthesising coping strategies used by individuals living with these conditions.

**Objectives:**

To identify, categorise and compare coping strategies employed by individuals with cancer, COPD, diabetes and coronary heart disease, and to examine the personal, familial and systemic factors shaping these adaptive responses.

**Methods:**

A systematic review of peer‐reviewed studies published in English between January 2010 and September 2024 was conducted across five major databases that include PubMed, CINAHL Complete, JSTOR, PsycINFO and ScienceDirect using a structured search strategy with Boolean operators and Medical Subject Headings (MeSH terms). Thematic analysis was employed to synthesise findings from the selected studies.

**Results:**

Eight core themes emerged: maladaptive stress strategies, maintaining ‘normalcy’, medication use, emotional factors, expanding social networks, therapeutic interventions, complementary therapies and the influence of religion, music and nature. These themes illustrate the multidimensional nature of coping with chronic illness. Problem‐focused coping (e.g., self‐management and goal setting), emotion‐focused coping (e.g., mindfulness and emotional support) and avoidance coping (e.g., denial and disengagement) were all prevalent. Social determinants such as healthcare access, family relationships and community support significantly influenced outcomes, with stronger social support linked to greater resilience and better adherence to treatment.

**Conclusion:**

Effective coping strategies for chronic disease management require a holistic approach that addresses both individual mechanisms and broader social and systemic influences. Interventions should improve healthcare accessibility, strengthen family and community networks and offer tailored psychological resources to foster resilience.

## Graphical abstract

This systematic review explores how individuals with cancer, chronic obstructive pulmonary disease (COPD), diabetes and coronary heart disease adapt to the complex challenges posed by chronic illness. The review was designed to identify, categorise and compare coping strategies across these conditions, while also examining how personal experiences, family dynamics and broader systemic factors shape adaptive responses. A comprehensive search of peer‐reviewed studies published between January 2010 and September 2024 was conducted using PubMed, Scopus and PsycINFO. Relevant articles were retrieved using predefined keywords and analysed thematically to generate a rich synthesis of adaptive coping strategies. The findings revealed eight major coping themes. These included the use of maladaptive stress responses such as avoidance or resignation; strategies to maintain a sense of ‘normalcy’ through daily routines and roles; strict adherence to medication and treatment regimens; emotional regulation techniques, including mindfulness, prayer and expressive outlets; the expansion of social networks and reliance on family, peer and community support; participation in formal therapeutic interventions; the use of complementary and alternative medicine practices; and the influence of cultural and spiritual resources such as religion, music and connection with nature. Coping approaches varied and often overlapped, ranging from problem‐focused strategies like goal‐setting and self‐management to emotion‐focused responses such as seeking reassurance or emotional expression and avoidance mechanisms like denial or social withdrawal. Importantly, the review found that access to healthcare, strength of family ties and supportive community environments played critical roles in enhancing psychological resilience and improving treatment adherence. This study makes a unique contribution to the literature by offering a multidimensional understanding of coping that integrates personal, emotional, cultural and systemic dimensions. It underscores the need for holistic public health interventions that address not only the psychological needs of individuals but also the social determinants and cultural contexts that shape chronic illness management.

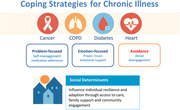

## 1 Introduction

Life presents numerous challenges. Among the most pressing are health and well‐being. The 20th century witnessed a significant epidemiological transition, characterised by a decline in infectious diseases and a corresponding rise in non‐communicable diseases (NCDs). Chronic diseases such as cancer, cardiovascular diseases, diabetes and respiratory disorders have become major global health concerns, exacerbated by risk factors such as poor dietary habits, physical inactivity and substance abuse [1, 2, 3]. As life expectancy increases worldwide, the incidence of chronic conditions is projected to rise, particularly among the ageing population, further burdening healthcare systems [4, 5].

The World Health Organisation (WHO) defines chronic diseases as conditions that persist for over 3 months, significantly impairing daily function and requiring long‐term management [6]. The global burden of chronic diseases is not confined to any region but is evident across high‐, middle‐ and low‐income countries. This trend is driven mainly by cardiovascular diseases, cancers, respiratory conditions and diabetes, which together account for a substantial proportion of global morbidity and mortality [7]. It is estimated that 80% of older adults live with at least one chronic illness, with 68.4% of Medicare beneficiaries managing multiple chronic conditions and 36.4% dealing with four or more [8, 9]. In clinical practice, approximately 70% of primary care visits are dedicated to managing chronic conditions [8, 10]. These diseases not only diminish daily functionality but also impose psychological and emotional burdens, leading to higher rates of anxiety, depression and social isolation. They further contribute to rising healthcare expenditures, polypharmacy, complex care coordination and persistent health disparities, all of which severely affect quality of life (QoL) [11].

**FIGURE 1:**
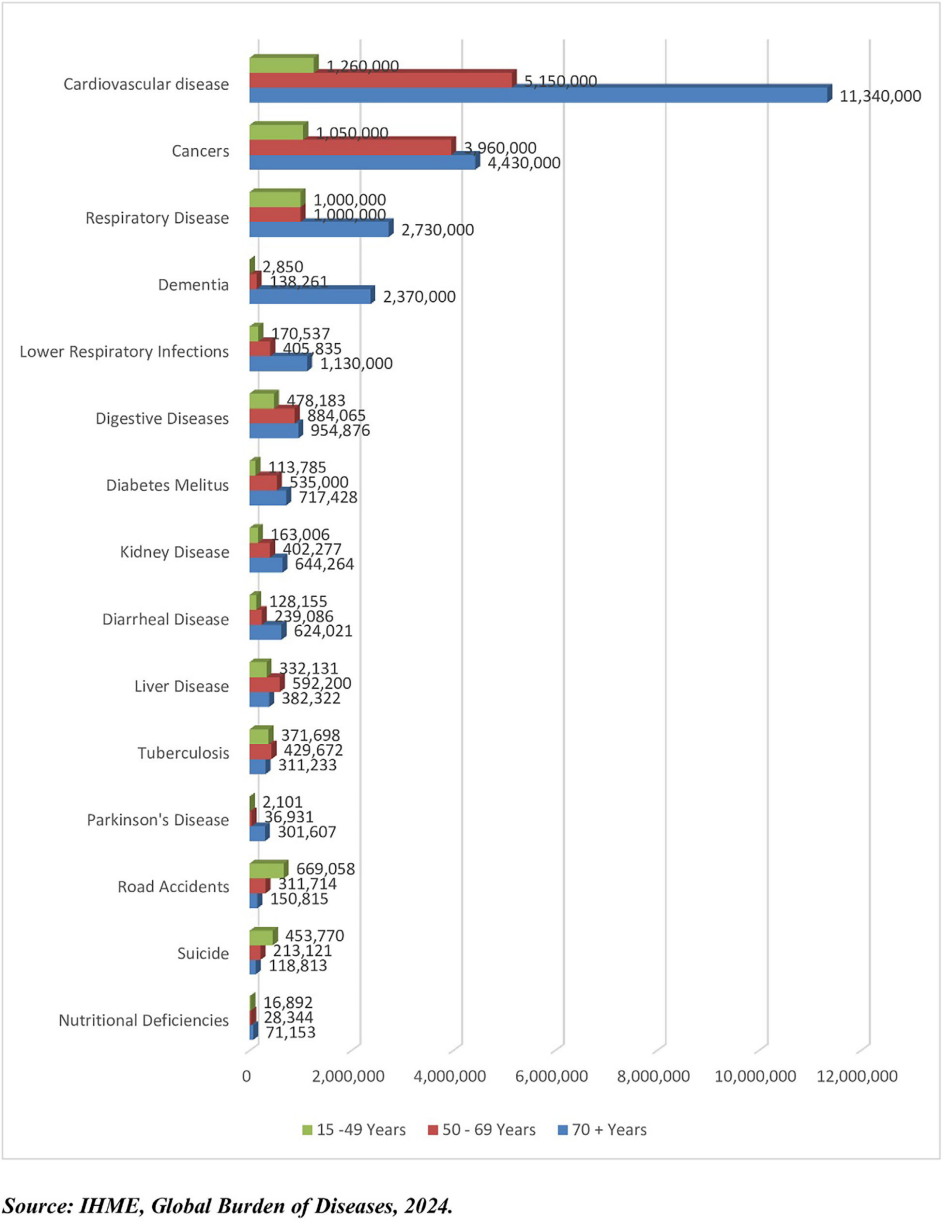
Combined causes of death for the age group 15–70+ years, 2024. *Source:* IHME, Global Burden of Diseases [13].

Despite the increasing prevalence of chronic diseases, there is limited research on the effectiveness of coping strategies and how they influence psychological well‐being, disease progression and overall adaptation. In response to this gap, recent studies on resilience have emphasised the need for individuals to develop adaptive mechanisms that enable them to withstand and flourish despite ongoing health challenges [12]. Chronic diseases are now responsible for more than half of all deaths worldwide, with coronary heart disease and stroke alone accounting for 15.2 million deaths out of 56.9 million in 2016. These mortality patterns have remained relatively stable over the last 15 years, underscoring chronic diseases’ persistent and severe impact on global health (Figure 1).

Figure 1 illustrates the leading causes of death among individuals aged 15 years and older, highlighting the dominance of chronic conditions across various age groups. The burden of these diseases is particularly pronounced among those aged 70 and above, where cardiovascular diseases and cancers remain the most prevalent causes of mortality. However, younger age groups are also significantly affected, particularly in low‐income countries, where communicable diseases such as HIV/AIDS and diarrhoeal diseases still contribute substantially to mortality rates.

Given the profound economic, social and psychological implications of chronic diseases, there is a growing need for comprehensive research on adaptive coping strategies. *Effective adaptation requires a multifaceted approach, integrating psychological resilience, social support and structured healthcare interventions*. Studies indicate that coping mechanisms are crucial in determining patient outcomes, with specific strategies fostering better psychological adjustment, whereas others may exacerbate distress and hinder treatment adherence [14].

Psychological distress, including depression and anxiety, is commonly observed in patients managing chronic conditions. Research has linked loneliness to poor health outcomes, exacerbating the severity of chronic illnesses and contributing to increased healthcare utilisation [15, 16]. Additionally, genetic predispositions, such as polymorphisms in the serotonin transporter gene, have been found to heighten the risk of depression in patients with chronic diseases [17, 18]. Therefore, understanding the interplay between biological, psychological and social factors in chronic disease management is essential for developing effective interventions promoting patient well‐being and resilience.

Coping mechanisms vary widely among individuals and can be categorised as problem‐focused, emotion‐focused or avoidance‐based strategies [19]. Problem‐focused coping involves direct efforts to manage the illness through treatment adherence and self‐care practices. Emotion‐focused coping, on the other hand, addresses psychological distress through mechanisms like mindfulness, social support and religious engagement. Avoidance coping, including denial or disengagement, often leads to poorer health outcomes [20, 21, 22]. Given chronic illnesses’ financial and social pressures, adaptive strategies are increasingly being explored as critical components of long‐term disease management [23].

However, defining and measuring adaptation remains a challenge in chronic disease research. Scholars have debated the conceptualisation of adaptation, highlighting the need for a more standardised framework to assess coping effectiveness [24, 25]. Some researchers classify coping strategies into positive (e.g., seeking social support and engaging in healthy behaviours) and negative (e.g., substance misuse and withdrawal) [26, 27]. In contrast, others distinguish between problem‐ and emotion‐focused strategies [28, 29]. Despite these varying frameworks, there is a consensus that coping mechanisms significantly influence treatment adherence, emotional stability and overall QoL for individuals with chronic illnesses [30].

Although previous reviews have examined coping strategies within various stress contexts, limited research synthesises adaptive mechanisms, specifically within the chronic disease population [31, 32]. This systematic review (SR) aims to bridge this gap by analysing coping strategies across four major chronic conditions: cancer, chronic obstructive pulmonary disease (COPD), diabetes mellitus and coronary heart disease.

The review seeks to provide a comprehensive synthesis of the coping strategies employed by individuals managing chronic diseases, focusing on internal and external adaptive mechanisms. It aims to examine the various coping strategies that individuals living with chronic conditions adopt in response to their health challenges. Additionally, it explores how internal coping mechanisms, including stress adaptation, routine maintenance, medication adherence, maladaptive responses and faith‐based practices, influence health outcomes. The study also investigates the role of external coping mechanisms, such as social network expansion, therapeutic interventions and complementary or alternative therapies, in shaping patient resilience. Furthermore, it synthesises existing research to generate evidence‐based insights into effective coping mechanisms that enhance the QoL for individuals dealing with chronic illnesses. In addressing these areas, the review seeks to provide valuable knowledge for healthcare practitioners, policymakers and researchers, supporting the development of targeted interventions that strengthen adaptive coping strategies and promote improved long‐term health outcomes.

## 2 Methods

The SR approach is highly effective for thoroughly analysing evidence on a specific topic [33]. In this review, we utilise the SR methodology to investigate chronic disease conditions and individuals’ adaptive strategies in response. Our final protocol was registered prospectively on Figshare with the https://doi.org/10.6084/m9.figshare.22892123. The SR method is valued for its meticulous and systematic approach, which aids in identifying emerging trends within the existing body of literature [34]. Additionally, the SR approach is adept at highlighting gaps in current knowledge and providing insights gleaned from comprehensive evaluations of evidence drawn from dedicated literature databases, all guided by precise research questions and methodologies [34]. To maintain transparency and ensure the rigour of our review, we followed the methodological guidelines outlined by the Preferred Reporting Items for Systematic Reviews and Meta‐Analyses (PRISMA 2020) [35]. This structured framework enhances the reliability and reproducibility of our findings, offering a robust approach to conducting SRs.

Employing the SR approach, we conduct an in‐depth exploration of chronic conditions such as cancer, COPD, diabetes mellitus and coronary heart disease. Our focus extends beyond mere description, enabling us to identify underlying trends and patterns in the existing literature. Through the application of standardised methodologies and specific research questions, we ensure the integrity and comprehensiveness of our review process. The SR approach offers several advantages, including systematically identifying key trends and gaps in the literature, contributing to a holistic understanding of the subject matter. This method allows the authors to move beyond superficial analysis and delve into the intricate aspects of chronic disease conditions and the adaptive approaches individuals use.

Therefore, the SR approach, conducted mainly according to the PRISMA 2020 guidelines, is an invaluable tool for systematically reviewing and analysing the evidence related to chronic disease conditions and adaptive strategies. Its methodical nature ensures that the findings are reliable and comprehensive, significantly contributing to the existing knowledge base in this field.

In addition, the authors implemented clear and precise inclusion criteria by adopting the population, intervention, comparison and outcome (PICO) framework, which is widely utilised in evidence‐based research. This approach aimed to enhance the transparency and rigour of the SR process, as detailed in Table 1.

**TABLE 1:** Population, intervention, comparison and outcome framework.

Key element	Criteria
Population	Individuals diagnosed with cancer, chronic obstructive pulmonary disease (COPD), diabetes mellitus or coronary heart disease
Intervention	Coping strategies utilised by individuals dealing with the mentioned chronic conditions
Comparison	Not applicable (as the focus is on understanding coping strategies rather than comparing interventions)
Outcome	Enhanced understanding of coping mechanisms and strategies among patients with cancer, COPD, diabetes mellitus and coronary heart disease

*Source:* Authors’ Construct [36] based on the PICO Framework.

All authors meticulously executed the SR process, with a strong emphasis on maintaining rigorous standards throughout the review. The authors conducted each review stage, including study selection, data extraction and quality assessment. This methodological rigour was essential in minimising potential biases and ensuring an objective analysis. In engaging directly with full‐text articles, the authors gained a deep understanding of the subject matter, enabling them to appreciate the contextual nuances and finer details of the studies under review. This comprehensive approach formed the basis for addressing any disagreements that arose.

When differences emerged during the review process, they were systematically resolved through collaborative efforts to achieve consensus. The resolution process involved thoroughly exploring shared perspectives and rational discussions, emphasising the importance of agreement as a practical solution and a moral obligation to maintain the integrity and objectivity of the SR.

Adhering to a methodological framework that prioritised independent analysis, thorough engagement with full‐text articles and deliberate consensus‐building, the authors ensured that the SR was conducted with the highest scholarly rigour. This approach reflected an unwavering commitment to producing credible and valid findings.

### 2.1 Literature Search Strategy

#### 2.1.1 Type of Study and Databases

This review focused on studies published between January 2010 and September 2024, incorporating both qualitative and quantitative analyses to ensure a thorough examination of the literature. The integration of these two approaches allows for a more comprehensive understanding, as each method compensates for the potential limitations of the other [37]. Qualitative research offers in‐depth insights into participants’ lived experiences and behaviours, although it sometimes lacks the objectivity and reliability necessary for establishing causality and generalising findings to broader contexts. On the other hand, quantitative research enables the generalisation of results to larger populations and provides precise answers to specific research questions. However, it has been criticised for neglecting individual perspectives, human experiences and behaviours, relying instead on measurable variables that may not fully capture these complex aspects.

To capitalise on the strengths of both methodologies while addressing their weaknesses, a comprehensive literature search was conducted across several leading databases, including PubMed, CINAHL Complete, JSTOR, PsycINFO and ScienceDirect [38]. These databases were carefully selected for their extensive interdisciplinary coverage and wealth of current scholarly literature. The chosen time frame was particularly relevant for capturing the evolving global trends in morbidity and mortality, which align with contemporary developments.

Each of these databases brings unique strengths to the review, complementing one another to enhance the study's overall robustness. For example, ScienceDirect is a crucial repository for scientific research, providing access to the physical and social sciences essential for examining the psychological dimensions of our study. JSTOR offers a vast collection of academic articles, books and primary sources across diverse disciplines, making it a valuable resource for scholarly inquiry. Meanwhile, CINAHL, the Cumulative Index of Nursing and Allied Health Literature, stands out as a crucial resource within the nursing field, with a comprehensive collection of 768 journals and indexing for over 5000 nursing and allied health journals.

Strategically leveraging these five distinctive databases, the review covers a broad range of medical, social and psychological research, focusing on how individuals adapt to chronic illnesses. This holistic approach provides a rich, multifaceted understanding of adaptive mechanisms in the context of long‐term health challenges.

### 2.2 Search Terms

Employing a meticulously crafted search strategy, we amalgamated pivotal keywords: ‘adaptive approaches’ and ‘patients with chronic disease’. This strategy was meticulously executed through the adept utilisation of essential Boolean operators: (‘cancer AND adaptive approaches’) OR (‘COPD AND adaptive approaches’) OR (‘diabetes mellitus AND adaptive approaches’) OR (‘Coronary heart disease AND adaptive approaches’). To further refine the precision of our search, we supplemented this strategy with the judicious integration of Medical Subject Headings (MeSH terms), a hallmark of scholarly precision. This step enabled a more comprehensive and nuanced exploration of the literature landscape. Furthermore, a linguistic criterion stipulated our focus solely on works published in English while ensuring the presence of abstracts or full‐text studies. This stringent approach aimed to capture a contemporary and substantively informative array of scholarly contributions.

### 2.3 Study Selection

#### 2.3.1 Criteria for Inclusion and Exclusion

This review focused on identifying adaptive approaches among individuals managing chronic illnesses, as detailed in peer‐reviewed publications. To maintain a high level of rigour, the inclusion criteria were strictly defined to include only studies published in English and approved by Institutional Review Boards, ensuring compliance with the ethical principles of the Helsinki Declaration [39].

On the other hand, certain exclusion criteria were applied. Studies were excluded if they were duplicate publications or if they appeared to examine adaptive strategies but did not provide substantial insight into this aspect during their investigation. Additionally, studies specifically addressing the adaptive responses of bereaved families following the death of individuals with chronic conditions were excluded. Non‐English studies and review articles discussing genetic disorders and acute illnesses were omitted. Finally, research focusing on the adaptive strategies of healthcare providers or caregivers rather than the patients themselves was also excluded from this review.

**FIGURE 2:**
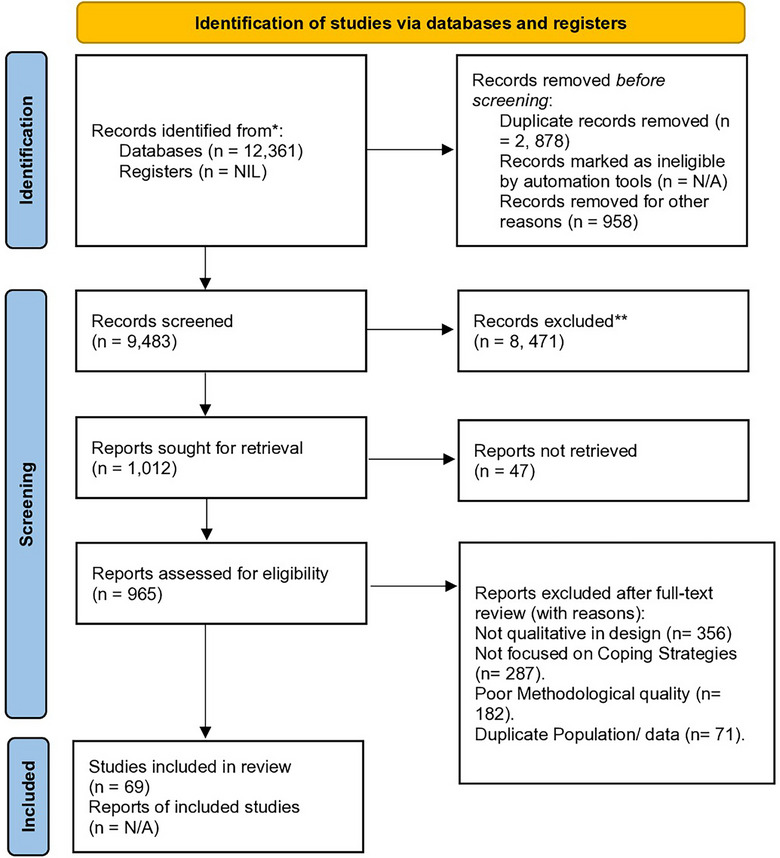
PRISMA 2020 chart showing the screening and selection procedure. Information extraction was methodically performed using a tailored data extraction template aligned with the study's objectives. Each publication underwent a manual data extraction process.

Figure 2 visually summarises the detailed article selection process, outlines the progression from abstract screening to full‐text review and provides a final selection of fifty‐four (54) studies, each meeting the rigorous inclusion and exclusion criteria set by the authors. These studies, centred on cancer, COPD, diabetes mellitus and coronary heart disease, formed the foundation for the systematic analysis.

## 3 Findings

Table 2 presents an overview of the critical characteristics of the studies included in this review. The fifty‐four (54) selected studies primarily concentrated on various chronic conditions, with the distribution as follows: cancer (33 studies), diabetes mellitus (12 studies), COPD (6 studies) and coronary heart disease (3 studies). Of these, thirteen (13) studies specifically examined adaptive approaches among children and adolescents, whereas the remaining forty‐one (41) focused on adults and older adults.

**TABLE 2:** General features and type of study included in review.

Feature	Percentage	Number (*n*)
**Type of chronic disease/Condition**		
Cancer	61.1	33
Diabetes mellitus	22.2	12
Coronary obstructive pulmonary disease	11.1	6
Coronary heart disease	5.7	3
**Methodology**		
Quantitative	64.8	35
Qualitative	35.1	19
**Type of article**		
Original research	100.0	54
**Design**		
Cross‐sectional	76.1	41
Longitudinal	15.0	8
Controlled experiment	9.0	5
**Regional distribution**		
Europe	44.4	24
North America	18.5	10
Asia	16.7	9
Australia	11.1	6
Sub‐Saharan Africa	5.7	3
Latin America	3.7	2
**Total**	**100.0**	**54**

*Source:* Authors’ Construct (2024).

In research methodology, thirty‐five studies (35) employed quantitative methods to explore adaptive strategies within their intervention programmes, treating these strategies as dependent variables. The other nineteen (19) studies adopted qualitative approaches. Notably, all fifty‐four (54) studies were original research articles. Regarding the study design, forty‐one (41) were cross‐sectional, eight (8) were longitudinal, and five (5) were controlled experiments.

The geographical distribution of the research was diverse: twenty‐four (24) studies originated from Europe, ten (10) from North America, nine (9) from Asia, six (6) from Australia, three (3) from sub‐Saharan Africa (SSA) and two (2) from Latin America.

### 3.1 Features of Studies Included in the Review

#### 3.1.1 Assessment of Study Quality and Risk of Bias

In adherence to the rigorous standards of SR methodology, this study incorporates a structured approach to assess the quality and risk of bias of the included studies. The following sections outline the methods employed for this critical aspect of the review process:

**Study selection criteria**:

- Inclusion and exclusion criteria were clearly defined to ensure relevance to the research question.
- Criteria were aligned with the aim of the SR, focusing on adaptive approaches among patients with cancer, COPD, diabetes mellitus and coronary heart disease.
- The rationale for each criterion was documented to justify study selection decisions, enhancing transparency and reproducibility.

**Search strategy**:

- The search strategy was described in detail, including databases searched, keywords used and additional search methods employed.
- The search timeframe and language restrictions were specified to ensure comprehensiveness and reproducibility.
- The search strategy was evaluated to minimise selection bias and enhance the robustness of the study selection process.

**Study identification and selection process**:

- The process for screening and selecting studies was outlined, including the number of reviewers involved and measures taken to resolve disagreements.
- Transparency and consistency in study selection procedures were assessed to minimise selection bias and ensure reliability.

**Data extraction**:

- The data extraction process was detailed, including variables extracted and methods used.
- Consistency in data extraction across included studies was ensured to minimise extraction bias.
- Pilot testing of the data extraction form was considered to refine its effectiveness and reliability.

**Assessment of study quality or risk of bias**:

- Appropriate tools or frameworks were selected for assessing study quality or risk of bias based on included study designs.
- The chosen assessment tool was systematically applied to each included study, evaluating key domains such as study design, type of study and database, population, intervention, comparison and outcome framework.
- Quality assessments were conducted independently by three reviewers to enhance reliability and minimise bias.
- Clear criteria for scoring or categorising study quality or risk of bias were provided to facilitate interpretation and comparison across studies.

**Data synthesis and interpretation**:

- Quality assessments were considered when synthesising and interpreting study findings.
- The impact of study quality or risk of bias on the overall results and conclusions of the SR was explored.
- Limitations or uncertainties related to the quality of included studies and their implications for review findings were discussed.

**Sensitivity analysis**:

To conduct a sensitivity analysis and assess the robustness of the review findings, we employed a systematic approach outlined as follows:

1. **Variations in study quality assessment**: We systematically varied the criteria for assessing study quality or risk of bias across included studies. This involved modifying the weighting or scoring of individual domains within the quality assessment tool. For example, we explored the impact of assigning different weights to critical domains such as study design, participant selection outcome measurement. Varying the criteria used for quality assessment, we assessed the consistency and robustness of the review findings.
2. **Exclusion of studies with high risk of bias**: On the basis of the quality assessment, we performed sensitivity analysis by excluding studies deemed to have a high risk of bias. This involved re‐analysing the data after removing studies with methodological limitations or concerns about bias. By comparing the results before and after the exclusion of high‐risk studies, we evaluated the influence of study quality on the overall conclusions of the review.
3. **Subgroup analysis**: We also conducted subgroup analysis based on study characteristics such as study design, geographic location and publication year. This allowed us to explore whether the findings were consistent across different subgroups and identify potential sources of heterogeneity.

**Criteria for scoring**:

The criteria for scoring study quality or risk of bias were based on established guidelines and frameworks relevant to the study designs included in the review. We utilised the following criteria as proof:

1. **Cochrane risk of bias tool**: For randomised controlled trials (RCTs), we employed the Cochrane risk of bias tool to assess critical domains such as random sequence generation, allocation concealment, blinding of participants and personnel, blinding of outcome assessment, incomplete outcome data, selective reporting and other sources of bias. Each domain was scored as low, unclear or high risk of bias based on predefined criteria.
2. **Newcastle–Ottawa scale (NOS)**: For observational studies, we used the **NOS** to assess methodological quality across three domains: selection of study groups, comparability of groups and ascertainment of outcomes. Each domain was scored on a scale from 0 to 9, with higher scores indicating higher methodological quality.
3. **Adapted criteria for mixed‐methods studies**: For studies employing mixed‐methods designs, we developed adapted criteria based on established guidelines for assessing the research's quantitative and qualitative components. These criteria encompassed methodological rigour, data integration and reporting transparency.

**Quality assessment process**:

The quality or risk of bias assessment was conducted systematically by independent reviewers, with discrepancies resolved through consensus. We provide proof of this process through the following steps:

1. **Training and calibration**: Reviewers underwent training and calibration sessions to ensure consistency in applying the quality assessment criteria. This involved reviewing a subset of studies together and discussing any discrepancies in scoring until a consensus was reached.
2. **Independent assessment**: Two or more reviewers independently assessed each included study for quality or risk of bias. To minimise bias, reviewers were blinded to each other's assessments.
3. **Consensus building**: Discrepancies in quality assessments were resolved through consensus discussions among reviewers. Any disagreements were documented and resolved through iterative discussions until consensus was achieved.
4. **Inter‐rater reliability**: Inter‐rater reliability was calculated to assess the agreement between reviewers’ assessments. This was done using statistical measures such as Cohen's kappa or intraclass correlation coefficients.

Providing detailed documentation of the sensitivity analysis, scoring criteria and quality assessment process, we demonstrate the rigour and transparency of our approach to assessing study quality and risk of bias. This comprehensive methodological framework enhances the reliability and credibility of the review findings, ensuring a robust synthesis of the available evidence.

**Reporting**:

- Results of quality assessments for included studies were transparently reported, including summary scores or ratings where applicable.
- Implications of study quality or risk of bias for interpretation and generalisability of review findings were discussed.
- PRISMA guidelines for reporting SRs were considered to ensure clarity, transparency and completeness in reporting the quality assessment process.

Adhering to this structured approach, the SR ensures a robust assessment of the quality and risk of bias of included studies, enhancing the reliability and credibility of the review findings.

**FIGURE 3:**
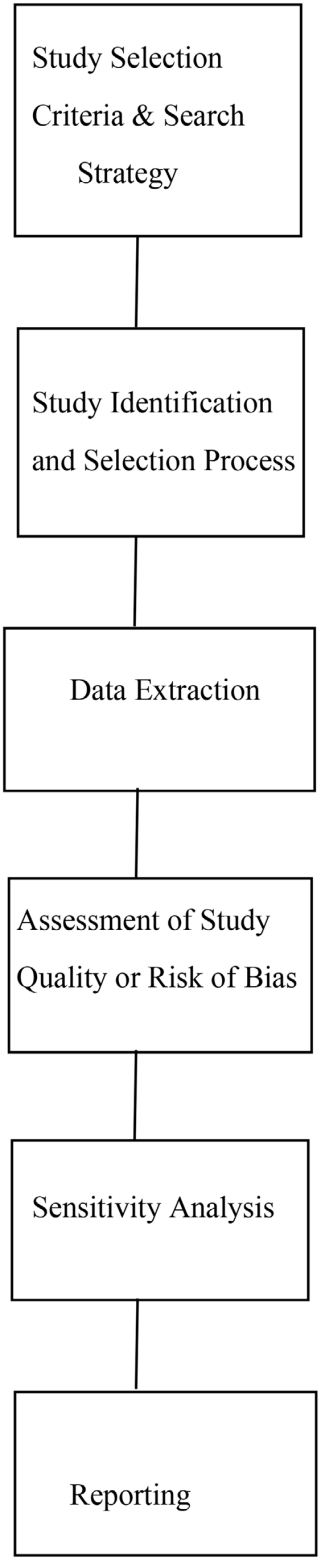
Sequential flow of steps involved in assessing study quality or risk of bias in a systematic review.

Figure 3 illustrates the sequential steps involved in assessing study quality or risk of bias in a SR, from defining study selection criteria and search strategy to reporting the findings.

### 3.2 Analysis and Coding

The final analysis included 54 studies, with each study's characteristics systematically coded and analysed. The findings revealed various adaptive strategies utilised by individuals, broadly categorised into two domains: external factors, such as social and environmental influences, and internal factors, including psychological and behavioural aspects. Through a thematic synthesis of the coded data, eight distinct themes emerged, collectively portraying the experiences of individuals managing chronic diseases. These themes were further grouped into the overarching internal versus external strategies dichotomy. These overriding themes form the primary tier of the thematic structure, encapsulating the perspectives presented in this review. The key themes identified are as follows:

1. Maintaining normalcy—Participants sought to preserve a sense of routine and identity through balanced nutrition, regular physical activity, fulfilling daily life tasks, managing family responsibilities and adjusting thought patterns to establish a new life rhythm.
2. Medical and behavioural management—Adherence to prescribed medications, attending medical follow‐ups and integrating recommended behavioural adjustments formed a core strategy for maintaining health stability.
3. Emotional/Psychological factors—Experiences of mental and physical fatigue, uncertainty and reduced peace of mind were common, with participants navigating complex emotional landscapes.
4. Stress (maladaptive) coping strategies—Some individuals adopted avoidance, denial or other maladaptive behaviours, which provided temporary relief but risked long‐term well‐being.
5. Expanding social worlds—Building and sustaining social networks, seeking support from family, friends, peers and community groups helped mitigate isolation and fostered resilience.
6. Complementary therapies (CTs)—Emotional‐ and meaning‐focused coping included practices such as herbal remedies, massage and other non‐conventional interventions to support mental and physical well‐being.
7. Therapeutic interventions—Use of psychological and pharmacological treatments, often in combination, addressed both physical symptoms and mental health needs.
8. Religion, music, nature and romanticism—Spiritual engagement through prayer, meditation, attendance at religious services, reading sacred texts and finding solace in nature or music offered comfort, meaning and emotional restoration.

### 3.3 Themes on Adaptive Strategies

#### 3.3.1 Organising and Basic Themes

**FIGURE 4:**
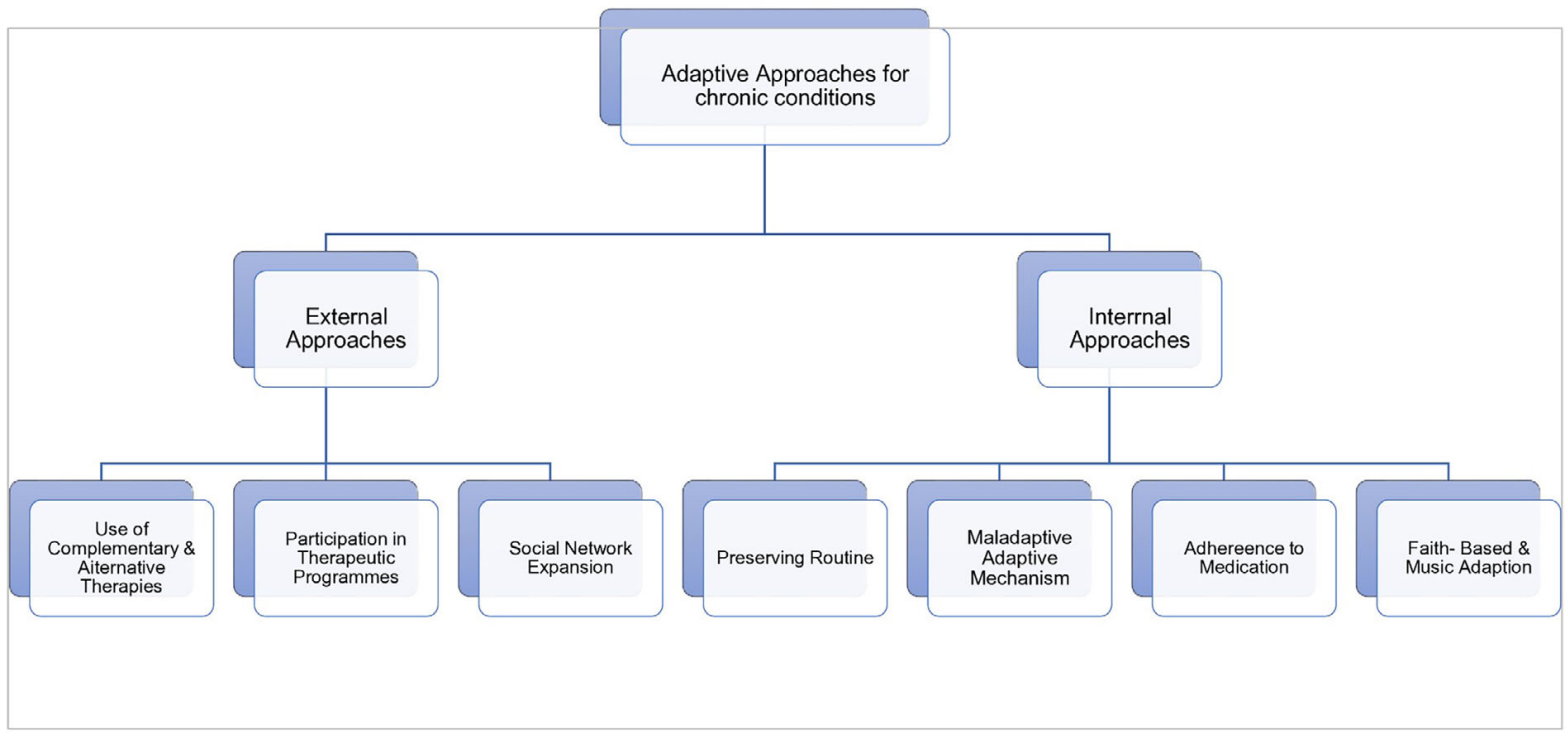
Classification of themes on adaptive approaches used by individuals living with chronic disease(s).

At the secondary tier of thematic organisation, organising themes arise to concisely capture the underlying viewpoints found in two or more extracted responses. These themes highlight the varied perspectives and lived experiences associated with the overarching theme. At the tertiary level, the transcripts draw foundational themes from direct quotations. These thematic layers were systematically classified within the internal and external appoaches framework, as illustrated in Figure 4.

### 3.4 Internal Strategies

#### 3.4.1 Maintaining ‘Normalcy’ With Family Relations

**FIGURE 5:**
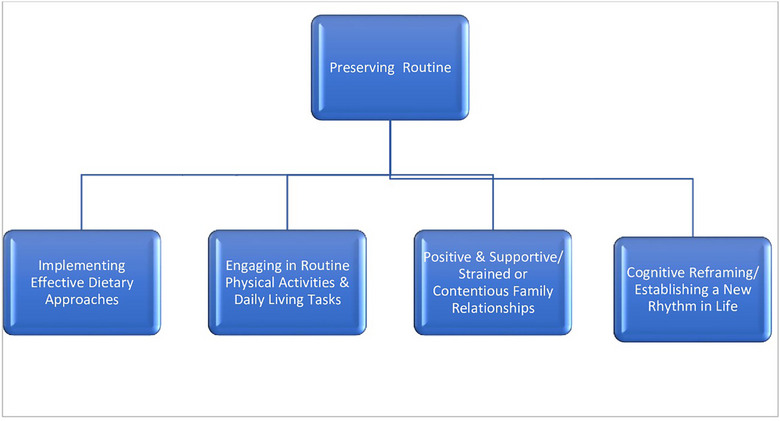
Sub‐themes under maintaining normalcy under internal coping strategies.

Once people develop a chronic disease, a range of barriers to life becomes a constant reality. This comes in the form of physical barriers, knowledge gaps about the disease, insufficient emotional support and financial burden in managing the disease [39, 40]. Amid these differences, people cope by trying to ‘maintain normalcy’. Maintaining normalcy refers to how individuals living with chronic conditions manage the impacts on everyday life, such as going to work, preparing food, sleeping arrangements and spending time with family and, if possible, entertaining activities. This theme of normalcy was reported in fifteen (15) studies, as shown in Table 2. Four sub‐themes were further identified under the theme of maintaining normalcy (Table 3). The four sub‐themes are as follows: exercise and dietary, good and supportive relations, difficult or quarrelsome relations, and seeking information by themselves (Figure 5).

**TABLE 3:** Articles on the theme of maintaining “normalcy”.

Author(s)/Year	Title	Objective(s)	Main Finding(s)
Roberts et al., 2018 [41]	A revised model for coping with advanced cancer.	To explore whether the Folkman and Greer theoretical model of appraisal and coping reflects the processes used by people with advanced cancer.	People with advanced cancer develop ways of “living around” and “living with” the disease. Coping fluctuated between positive and negative responses.
Scrignaro et al., 2011 [42]	The combined contribution of social support and coping strategies in predicting post‐traumatic growth.	To investigate the role of social support and coping strategies in enhancing post‐traumatic growth (PTG) in cancer patients.	Psychological support and active engagement helped improve PTG among cancer patients.
Yasui‐Furukori et al., 2019 [43]	Coping behaviours and depressive status in individuals with type 2 diabetes mellitus	To examine associations between coping behaviours and depressive symptoms.	Avoidance, emotional expression, and cognitive reframing were linked to depressive symptoms.
Yadav et al., 2018 [44]	Self‐management and patients’ activation in COPD patients.	To identify benefits and gaps in self‐management interventions.	Patient‐preferred coping strategies varied, with no uniform best method.
Gaston‐Johansson et al., 2013 [45]	Multimodal coping strategy program on QOL among breast cancer patients	To examine the effectiveness of a self‐management coping strategy program (CCSP).	CCSP improved quality of life; viewed as beneficial.
Vikki Knott et al., 2012 [46]	Understanding the cancer‐coping process: A grounded theory approach	To examine communication and social contexts of coping.	The social environment significantly shapes cancer‐coping experiences.
Koerich et al., 2013 [47]	Myocardial revascularization: Coping strategies with CHD and surgery	To understand the strategies used during coronary heart disease and surgical recovery.	Family support, spiritual resources, and rehab programs helped patients.
Bazzazian & Besharat, 2012 [48]	Adjustment to type I diabetes based on attachment and coping theory	To develop a model of adjustment to diabetes.	Positive illness perception and task‐oriented coping improved adjustment.
Jibb et al., 2018 [49]	Children's experiences of cancer care: a systematic review	To synthesize the experiences of children with cancer.	Patients longed for normalcy but developed resilience through support.
Ghiggia et al., 2017 [50]	Psychological distress and coping in nasopharyngeal cancer	To explore coping and QoL among NPC patients.	High distress is linked to dysfunctional coping, like hopelessness.
Religioni et al., 2017 [51]	Strategies for coping with pain in cancer	To assess how site and socioeconomics influence coping.	Coping strategy mediates pain perception and QoL.
Kanne Sarenmalm et al., 2017 [52]	Sense of coherence and QoL in breast cancer	To test the SOC theory among women with breast cancer.	Strong SOC predicted adaptive coping and higher QoL.
Ghodraty‐Jabloo et al., 2015 [53]	Coping with the long‐term effects of AML	To explore coping among leukemia survivors.	Mind distraction and social reassurance were key coping strategies.
Chatrung et al., 2015 [54]	Wellness and religious coping in Thai CKD patients	To develop interventions for CKD patients.	Family, social support, knowledge, and faith shaped coping.
Mosher et al., 2015 [55]	Coping in advanced lung cancer patients and caregivers	To identify physical and emotional coping strategies.	Routine, family support, and cognitive reframing were common.
Cheng et al., 2019 [56] (China)	Patients' experiences of coping with multiple chronic conditions	To explore the experiences of how Chinese adults cope with multiple chronic conditions in everyday life	Emphasis on preserving social roles and routines to maintain psychological balance and a sense of normalcy.
Zhang et al., 2024 [57] (China)	Disease coping experiences of pancreatic cancer patients and their spouses	To explore dyadic coping between patients and spouses	Adjustment of household roles and emotional exchange helped restore daily normalcy.
Zhang et al., 2024 [57] (Malaysia)	Lived experience of resilience in chronic disease among adults in Asian countries	To explore lived resilience experiences in Asia.	Maintenance of normal life was framed through family roles, spiritual grounding, and emotional reframing.
Senanayake et al., 2024 [58] (Sri Lanka)	Experiences of CKD patients and caregivers on haemodialysis	To explore the psychosocial impact and coping among CKD patients	Coping involved strict routines, redefining purpose, and sustaining identity through work and family.
Babić et al., 2020 [59] (China)	The experience of living with coronary heart disease among Chinese patients	To explore the lived experiences of CHD patients in urban China	Patients minimized disruptions through emotional self‐regulation, family dependence, and maintaining outward normalcy.
Kalantar‐Zadeh et al., 2021 [60] (Iran)	Coping with chronic cardiovascular disease in Iran	To understand psychosocial coping with CVD	Strategies involved spiritual acceptance, minimizing stressors, and reintegration of faith into daily life.
Zhang et al., 2024 [57] (China)	Coping strategies among older adults with multimorbidity	To understand coping practices for multiple chronic conditions	Social engagement and maintaining small daily rituals were used to restore a sense of stability and purpose.
Kragting et al., 2024 (Netherlands)	Illness perceptions in people with chronic neck pain.	To explore illness perceptions and their origins.	Participants adapted daily routines to manage persistent pain and preserve normalcy.
Zehrung & Chen, 2024 [61] (UK)	Self‐Expression and Sharing around Chronic Illness on TikTok	To investigate the use of TikTok for self‐expression and support	Shared experiences promoted awareness and normalized chronic illness.
O'Leary et al., 2020 [62] (UK)	Privacy risks in online health support for IBD	To explore IBD patients' views on social media use	Patients balance online support benefits with privacy concerns to sustain normal life.
Bulathwatta et al., 2024 [63] (Poland)	Experiences of individuals with CKD and caregivers	To explore psychosocial experiences during haemodialysis	Participants emphasized maintaining daily routines to preserve normalcy.
O'Connor et al., 2023 [64] (Ireland)	Coping with incurable cancer: A metasynthesis	To synthesize coping experiences in hospice care	Daily activities and identity preservation helped patients maintain normalcy.
Smith et al., 2022 [65] (Germany)	Caring for the self in chronic illness	To examine self‐transformation during illness	Patients redefined their identity and used self‐care to maintain normalcy.
Johansson et al., 2024 [66] (Sweden)	Parents' experiences caring for children with dermatitis	To explore how families cope with childhood illness	Parents integrated care into daily life to sustain normal family dynamics.
Müller et al., 2021 [67] (Switzerland)	Coping strategies in chronic heart failure patients	To identify coping in chronic heart failure	Normalcy is maintained through routine, social activity, and treatment adherence.

### 3.5 Incorporating Appropriate Nutritional Management/Activity Into Daily Life

An important category of coping was physical activity, as participants mentioned that it is important for changes in health behaviour. Patients living with chronic diseases are advised to keep performing daily walks or join fitness groups, but they must not lose sight of their cardiac and clinical conditions. Other activities that can be undertaken are yoga and walking activities to give them a sense of relief. For example, one patient reported that

I started walking and exercising … I used to get winded doing the steps up one floor. Now I am up to four steps. [54]

Patients also reported that relaxation exercises help them to cope better. For example, one patient mentioned that relaxation exercises on chronic diseases and face‐to‐face sessions were taught [68]. They were mainly breathing exercises designed to conquer anxiety and also to help develop feelings of security and confidence. Another patient mentioned that

I certainly don't lie in the chair or on the couch all day. I … do the dishes and sweep do some of the washing, enough to keep me where I don't feel worthless (62‐year‐old male lung cancer patient. [56]

An additional significant coping strategy observed among individuals with chronic diseases involves the adoption and sustained adherence to a healthy lifestyle. This encompasses practices such as reducing sugar consumption, prioritising fibre‐rich foods, opting for low‐calorie meals and consistently incorporating specialised dietary choices into their lifelong routines. Participant perspectives on the aspect of self‐care maintenance are exemplified in the following statements [47, 50].

My self‐care was to limit my diet and drink less than one liter of water per day. I have to be aware of everything that affects my health, such as diet, stress, medication, rest, and wound care.

I cook food that is low in salt and fat. My food also includes steamed or boiled white meat. I avoid fruits and vegetables such as bananas and avocados. I have not missed an HD appointment or skipped any of my medication since I started my treatment. I exercise regularly. My goal is to become healthy so that I can be prepared for my kidney transplant. I do not want any complications. I observe my symptoms and monitor my blood sugar level and blood pressure every day.

I eat food that is appropriate for my health condition. I have to try to eat enough, or else I will grow fatigued. Sometimes I ate fried fish or meat when I grew tired, or steamed fish and boiled meat. Earlier, when I was first diagnosed with CKD, I could not control my diet. But after 4 months, I adapted and learned how to live with the disease.

For individuals who underwent kidney transplants, their objective post‐transplant was to uphold both their overall health and the well‐being of the transplanted kidney.

My life depends on medication. I have to take pills for the rest of my life. I exercise daily. Taking care of my health is my duty in order to maintain the functions of my kidneys. I had a kidney transplant 10 years ago. Hopefully, I can live with this kidney up to 15 years.

I cook food that is low in salt and fat. My food also includes steamed or boiled white meat. I avoid fruits and vegetables such as bananas and avocados. I have not missed an HD appointment or skipped any of my medication since I started my treatment. I exercise regularly. My goal is to become healthy so that I can be prepared for my kidney transplant. I do not want any complications. I observe my symptoms and monitor my blood sugar level and blood pressure every day.

### 3.6 Good and Supportive Family Relations

Other studies reporting on the good and supportive relations revealed that, when family members are around the patient during hospitalisation, it motivates the patients, and they are able to go through procedures such as surgery. When family members are not present, patients do not have the energy to undergo surgery. The patient, at the crucial time of surgery, feels a sense of support and security to navigate through this difficult process. Some patients reported that

It means everything! I have my kids to help me. It is better to be helped by my children than by the hands of a stranger. [45, 47]

The support the family offers makes the patient confident to accept the surgery while, at the same time, reducing his/her anxiety and stress. One patient reported that

Without them (the family), I would have run away because it is very sad to stay in one place and not have the support of the family. [46]

In the view of health professionals, the friendship within the family is like a rock when a patient is going through the surgical process because it calms the patient. As a result, the inclusion of the family becomes integral within the pre‐ and post‐operative nursing directives. This involvement spans from the period of surgical preparation to the phases of recuperation and rehabilitation, extending even beyond discharge. In almost all situations, most family members try to engage in conversation to calm the family members and patients as well. The professionals advise this encouraging and supportive atmosphere at the time of the orientation [47]. In their investigation of myocardial revascularisation, Koerich et al. [47] identified a distinct construct where ‘sensing the presence and receiving support from the family’ provided patients with the empowerment needed to undergo the surgical procedure. This phenomenon is evident in the subsequent statement:

They [the family members] were very important; […] made me happier. (F9)

The patient, given his state of fragility after the indication of cardiac surgery, needs the family members in the sense of searching for security and support in a strange environment, different than the usual.

It means everything! I have my kids to help me. It is better to be helped by my children than by the hands of a stranger. (E5)

The family supports smoothies and the acceptance of surgery, with a consequent decrease in the anxiety and stress generated at the moment of surgery indication.

Without them [the family], I would have run away because it is very sad to stay in one place and not have the support of the family (F9).

### 3.7 Difficult or Quarrelsome Relations

For the sub‐theme on difficult or quarrelsome relations, the review identified that families who exhibit cantankerous tendencies lead to a compromise in the support net for the patient. But there are times the intervention of health professionals before the hospitalisation process helps mend broken families. In the absence of these developments, the patient's emotional balance is altered, creating further anxiety. For example, in one study, it was reported that there is

No use in bringing problems (family), no good, ‘because otherwise it will make the patient more anxious, more worried. [49]

#### 3.7.1 Cognitive Restructuring/Developing a New Cadence to Life

The final sub‐theme under maintaining normalcy, which is ‘seek information by themselves’, relates to patients’ desire to seek information aimed at gaining more insights into their condition and how to best manage it. Such information is usually obtained from workshops, magazines, newspapers and so forth [46, 54, 55, 69, 70, 71]. These studies on seeking information about the cancer that is afflicting them and as a means of maintaining normalcy [46, 54, 72] further showed that there is a positive correlation between family cooperation and improved care after the period of hospital discharge, which improves the recovery process. In their seminal research focusing on the adjustment model to Type 1 diabetes rooted in attachment, coping and self‐regulation theories, Bazzazian and Besharat [48] revealed that cultivating positive perceptions of illness and employing task‐oriented coping strategies serve as predictive factors for improved adaptation to diabetes. Consequently, these findings substantiated both theoretical foundations and empirical substantiation for the efficacy of attachment styles in addressing the challenges of chronic illnesses. This discovery holds potential for shaping preventive policies, identifying high‐risk patients with inadequate adaptation and formulating targeted psychological interventions. Moreover, patients and their families frequently confront alterations in family dynamics and lifestyle post‐hospital discharge, thereby introducing new stressors. The pursuit of equilibrium during this adjustment phase may instigate additional pressure, potentially leading to diminished social interactions and engagement (Table 4).

**TABLE 4:** Reference to themes.

Global theme	Organising theme	Basic theme or sub‐theme	Reference/Citation
**Internal strategies**	Maintaining normalcy	Incorporating appropriate nutritional management	[73, 74, 75, 76, 77, 78, 79]
		Physical activity/Activity daily life (ADL)	[42, 46, 49, 68, 73, 75, 77, 78, 79, 80, 81, 82]
		Family burdens	[45, 48, 49, 50, 68, 73, 77, 78, 79, 82, 83, 84, 85]
		Cognitive re‐structuring/Developing a new cadence to life	[46, 48, 49, 55, 71, 74, 75, 79, 82, 83, 84, 86, 87, 88, 89, 90, 91]
	Medical and behavioural management	Medication use/Attending follow‐ups	[41, 43, 45, 48, 49, 55, 68, 72, 74, 75, 80, 83, 92, 93, 94, 95, 96]
	Emotional/Psychological factors	Mental and physical fatigues/Uncertainties/Lack of peace of mind	[34, 41, 42, 43, 44, 45, 48, 51, 55, 56, 70, 71, 72, 73, 74, 80, 84, 86, 89, 92, 97, 98, 99, 100, 101, 102, 103]
	Stress (maladaptive) coping strategies	Maladaptive coping strategies	[41, 42, 44, 45, 48, 51, 55, 71, 72, 73, 80, 92, 96, 97, 100, 101, 102, 103, 104]
**External strategies**	Expanding social worlds	Seeking social support	[42, 46, 47, 49, 50, 55, 56, 68, 69, 70, 71, 72, 74, 75, 76, 81, 84, 105, 106, 107, 108, 109, 110, 111, 112]
	Complementary therapies (CTs)	CTs used for emotion‐focused coping	[41, 48, 55, 77, 78, 79, 80, 85, 104, 113, 114, 115, 116]
		CTs used for meaning‐focused coping	[45, 50, 71, 75, 78, 84, 87, 105, 109]
	Therapeutics interventions	Psychological/Pharmacological interventions	[45, 47, 48, 49, 50, 51, 55, 68, 69, 71, 72, 73, 75, 80, 81, 84, 88, 89, 90, 92, 93, 95, 102, 103, 104, 109, 117, 118]
	Tendency towards religion	Prayer	[48, 50, 78, 79, 88, 95, 100, 119]
		Meditation	[48, 78, 79, 82, 87, 88, 89, 90, 91, 95, 100, 114, 119, 120]
		Attendance at religious services	[48, 69, 71, 72, 78, 79, 82, 88, 90, 91, 119, 120]
		Reading sacred texts	[48, 50, 68, 73, 75, 78, 79, 87, 88, 89]
		No miracle	[68, 75, 80]
	Tendency towards natural music/Romanticism	Health promoting gardens/Bird songs/Wind	[78, 79, 86, 90, 105, 119]

### 3.8 Medication Use/Attending Follow‐Ups

Taking medicine was a common denominator in about half of the studies reviewed [46, 50, 54, 70, 43, 48, 51, 71, 72, 73, 74, 92]. Patients reported using medication and oxygen as a common way of managing chronic conditions because it lessens the physical and psychological symptoms. Caregivers undertake vigilant supervision of patients to ensure meticulous adherence to medication regimens, aiming to optimise their QoL. Consistent with this approach, night administration of oxygen was implemented for patients to alleviate sensations of breathlessness, fatigue and anxiety. This sentiment is echoed by a patient's testimony:

I started getting an anxiety attack and reached a point where I felt like I couldn't breathe anymore … so my doctor prescribed oxygen. [41] (63‐year‐old male patient)

Similar sentiments were echoed in other studies [41, 43, 48, 74, 75, 80, 83]. In one study by Li et al. [75], coping strategies employed by Chinese children undergoing cancer hospitalisation exhibited no variation based on gender or diagnosis. However, distinctions emerged based on age, wherein younger children demonstrated a tendency for reduced utilisation of problem‐focused coping and an inclination towards heightened employment of emotion‐focused coping mechanisms compared to their older counterparts. Notably, the comprehensive outcomes of this study revealed that among these Chinese patients, 30% adopted problem‐focused coping strategies, whereas a majority of 70% resorted to emotion‐focused coping strategies. Some of the problem‐focused strategies were treatment with moderate or highly melodic chemotherapy, whereas the emotion‐focused strategies were distraction, emotional regulation, wishful thinking and watching television [93]. Finally, the complexities associated with managing a multitude of medications, coupled with the subsequent financial burdens, emerged as significant concerns. Several studies highlighted the obstacles related to medication adherence (Table 5). Again, intricate schedules and apprehensions about potential adverse effects were also reported [48, 80, 94, 95]. One patient reported that

I get nervous if I have to take a couple of different medications at the same time …. [80]

**TABLE 5:** Articles on the theme of medication use.

Author(s)/Year	Title	Objective(s)	Main Finding(s)
Yisha Y. and Kerry A.S. 2015 [95]	Communication avoidance, coping, and psychological distress of women with breast cancer	This study examined the relationship between communication avoidance of cancer‐related topics with psychological distress, and the mediating role of coping strategies, in women with breast cancer.	The results of this study suggested that couples coping with cancer have more difficulties discussing emotionally valenced topics related to disease progression and death, sexuality, and feelings than more practical concerns such as cancer treatment.
Ghodraty‐Jabloo et al., 2015 [53]	Keep your mind off negative things: Coping with the long‐term effects of acute myeloid leukemia (AML)	The purpose of our study was to explore how AML survivors describe their longer‐term physical and psychosocial well‐being and how they cope with these challenges.	A range of emotion‐ and problem‐focused coping strategies was reported. Keeping one's mind off negative things through engaging in formal work or informal activities and seeking control were the two most commonly used coping strategies. Seeking social support for reassurance was also common. Problem‐focused strategies were frequently described by the ongoing treatment group to manage treatment side effects.
Gaston‐Johansson et al., 2013 [45]	This review is an attempt to identify the benefits and knowledge gaps in self‐management and patient‐activation positions in COPD self‐management interventions.	This study aims to examine the effectiveness of a self‐management multimodal comprehensive coping strategy program (CCSP) on quality of life (QOL) among breast cancer patients 1 year after treatment.	The CCSP improved QOL for patients at 1‐year follow‐up. Patients overwhelmingly reported that CCSP was beneficial. The CCSP as an effective coping intervention has potential as a self‐management program for breast cancer survivors.
Roberts et al., 2018 [41]	A revised model for coping with advanced cancer. Mapping concepts from a longitudinal qualitative study of patients and carers coping with advanced cancer onto Folkman and Greer's theoretical model of appraisal and coping	To explore whether the Folkman and Greer theoretical model of appraisal and coping reflects the processes used by people living with advanced cancer	This study revealed that people with advanced cancer develop ways of “living around” and “living with” that which comes with the cancer. The coping process for people with advanced cancer appears non‐linear, fluctuating between positive and negative issues, additional events, threats, harms, and challenges.
Préau et al., 2013 [83]	Two years after cancer diagnosis, what is the relationship between health‐related quality of life, coping strategy, and spirituality?	This study aimed to analyse the relationship between spirituality, coping strategies, and health‐related quality of life (HRQL) among a large representative sample of patients two years after cancer diagnosis.	The results of this study highlight how spirituality may be a valuable source of comfort during cancer treatment and consequently may play an important role in the management of one's disease. It especially questions about the role of religious actors in providing the support that patients expect as well as helping them to cope with the disease.
Rassart et al., 2016 [105]	Coping with type 1 diabetes through emerging adulthood: Longitudinal association with perceived control and hemoglobin A1c	To examine how perceived personal control, coping, and HbA_1c_ relate to one another over time.	Over the course of the study, patients showed a worsening of glycaemic control and a decrease in perceived personal control. A longer illness duration has indeed been associated with poorer glycaemic control, more severe hypoglycaemic events, and poorer dietary self‐care in individuals with type 1 diabetes.
Chatrung et al., 2015 [54].	Wellness and religious coping among Thai individuals living with chronic kidney disease (CKD) in Southern California.	To assist healthcare professionals in developing and implementing educational and supportive interventions for patients with CKD.	The results showed an interrelationships of four (4) emerging categories to have a direct impact on chronic disease‐ family relations, social support, knowledge/awareness and religious coping affect self‐care and how one manages one's life.
Chatrung et al., 2015 [54]	Wellness and religious coping among Thai individuals living with chronic kidney disease (CKD) in Southern California.	To assist healthcare professionals in developing and implementing educational and supportive interventions for patients with CKD.	The results showed an interrelationships of four (4) emerging categories to have a direct impact on chronic disease‐ family relations, social support, knowledge/awareness and religious coping affect self‐care and how one manages one's life.
William Li et al., 2012 [121]	Coping strategies used by children hospitalised with cancer: an exploratory study.	The purpose of this study was to examine the coping strategies used by Chinese children hospitalised with cancer, an area of research that is under‐represented in the existing literature.	The coping strategies used by Chinese children hospitalised with cancer did not differ according to gender and diagnosis, but only according to age, with younger children using less problem‐focused and more emotion‐focused coping strategies than older children. The overall results indicated that 30% of these Chinese patients used problem‐focused coping strategies, while 70% used emotion‐focused coping.
Mosher et al., 2015 [55]	Coping with physical and psychological symptoms: a qualitative study of advanced lung cancer patients and their family caregivers	This study aimed to identify strategies for coping with various physical and psychological symptoms among advanced, symptomatic lung cancer patients and their primary family caregivers.	Patients and caregivers reported maintaining a normal routine and turning to family and friends for support with symptom management, which often varied in its effectiveness. Whereas support from health‐care professionals and complementary and alternative medicine was viewed favorably, reactions to Internet and in‐person support groups were mixed due to the tragic nature of participants’ stories. Several cognitive coping strategies were frequently reported (i.e., changing expectations, maintaining positivity, and avoiding illness‐related thoughts) as well as religious coping strategies.

### 3.9 Emotional/Psychological Factors

**FIGURE 6:**
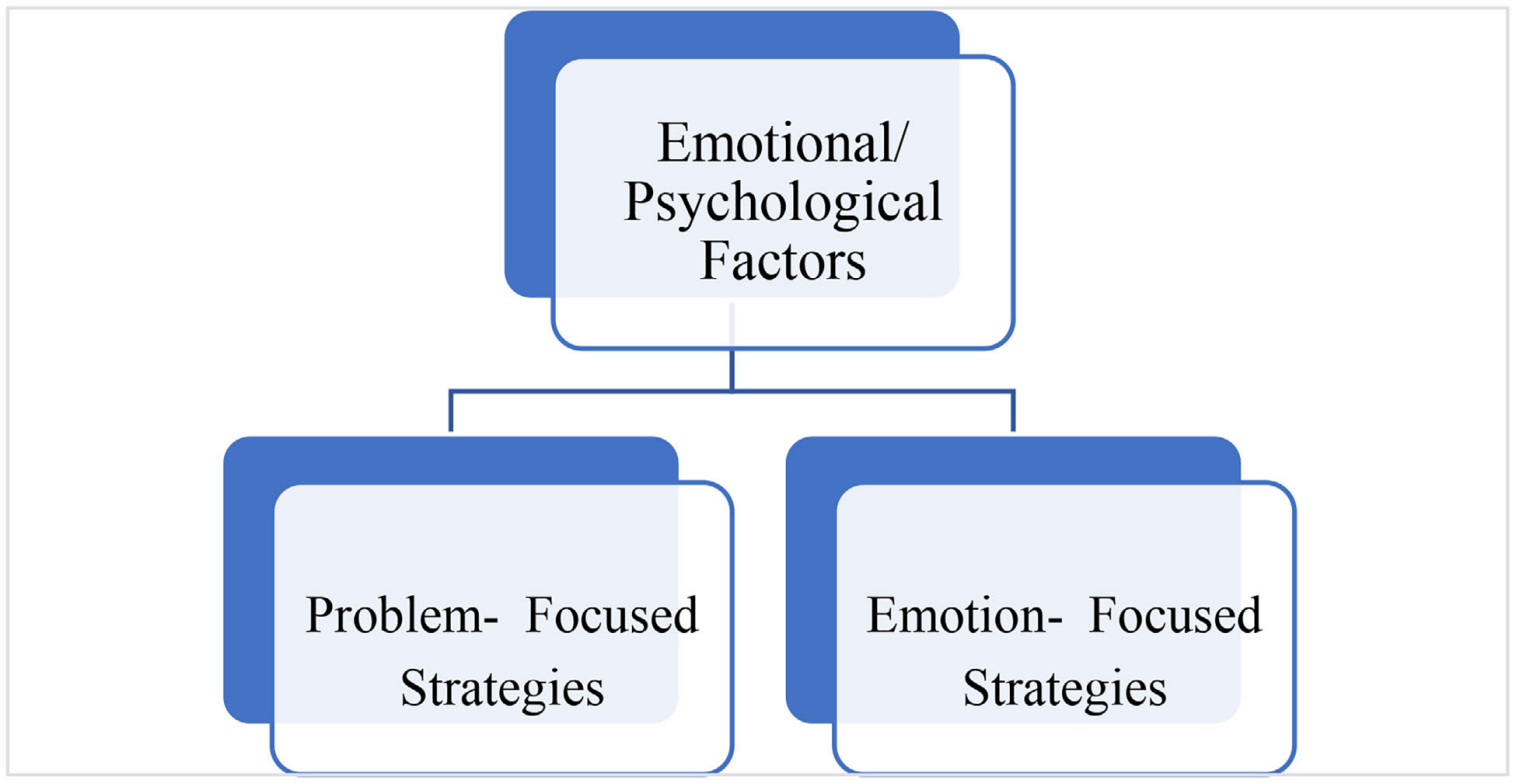
Sub‐themes under emotional/psychological factors under internal coping strategies.

An emotion is a complex psychological state that involves three distinct components: a subjective experience, a physiological response and a behavioural or expressive response [96]. In trying to define emotion, researchers have attempted to identify and classify the different types of emotions. Psychologist Paul Eckman [98] postulated the existence of six fundamental emotions that exhibit universality across diverse human cultures: fear, disgust, anger, surprise, happiness and sadness. Subsequently, in 1999, Ekman expanded this roster to encompass additional basic emotions, including, but not limited to, embarrassment, excitement, contempt, shame, pride, satisfaction and amusement. In the context of coping, chronic disease is like an albatross or, better still, an encumbrance on one's life. In coping with chronic conditions, positive and expressive emotional responses could facilitate favourable coping strategies, whereas negative and repressive emotional responses would yield negative adjustment [99]. The expansive works on coping with chronic disease suggest that the incidence of avoidance, diverting attention and catastrophising (extreme pessimism, imagining the worst situation) tends to lead to poor results, whereas the expression of positive emotions tends to yield a positive correction process [92]. A total of twenty‐six (26) articles in this review focused on emotional factors that are internal to the patient and spring from his/her inner drive or motivation to enhance the correction process. This corrective process could either bring about healing, improved QoL or a better way of managing the disease progression in patients and ultimately help with the coping process. The studies included in this review mainly distinguished between two emotional factors, namely, problem‐ and emotion‐focused coping strategies (Figure 6). Patients who use problem‐focused coping strategies exhibit lower anxiety as well as depressive symptoms. Hence, when patients adopt problem‐focused, it results in reduced anxiety and a lower depressive status. On the other hand, patients with chronic conditions who focus on emotion‐focused coping strategies tend to experience increased anxiety and depressive symptoms in patients and their spouses. Again, QoL is also decreased at the mental level [72, 84, 86, 92].

The articles reviewed revealed that coping strategies affect the patient's world view of pain and their capacity to endure through their daily activities [48, 50, 54, 70, 71, 72]. Furthermore, an array of elements, including adverse emotional states (such as anger, avoidance/denial, depression and anxiety), contextual environmental factors, social influences and cultural dynamics, converge with the biological dimension of pain. This convergence intricately shapes an individual's ability to acclimatise to and address pain stimuli [48, 50, 70, 71, 72, 73, 43, 105]. Several studies explored the association between lower QoL and reduced life satisfaction, revealing a nexus of mechanisms. These mechanisms encompass tactics such as diverting attention by attending to alternative responsibilities, re‐evaluating the perception of pain's significance, engaging in catastrophising by envisioning the direst scenarios, overlooking the pain, resorting to prayer or hope, invoking coping sub‐statements marked by the conviction of pain management feasibility and adopting behavioural strategies, including heightening activity levels within their daily routines [43, 70]. For example, one patient, on a positive note, said that

I've heard from people that you can worry yourself sick, and I just don't want to do that. I try to stay positive (50‐year‐old male cancer patient). [34]

Other studies also stated that women with a strong sense of coherence (SOC) experience fewer stressful events and days [55, 73]. Some studies also report a positive relationship between illness representations and coping behaviours of patients with cancer [74]. Moreover, positive illness acceptance mixed with task‐oriented coping strategies results in better adjustment to diabetes [48]. Additionally, the relationship that exists among health workers, family relations, social support and religious groups improves the patient's self‐care, which improves physical, psychological, social and spiritual aspects of the life of patients with chronic conditions [50]. Numerous patients articulated a variety of cognitive coping strategies in their accounts, encompassing shifts in expectations, the cultivation of a constructive viewpoint and deliberate avoidance of illness‐related ruminations. Patients exhibited an inclination to recognise the constraints imposed upon their activities, emphasising the imperative of acceptance. Additionally, they displayed a propensity to recalibrate their anticipations for the future and grapple with the inexorability of the disease trajectory and mortality. A subset of patients detailed their endeavours to supplant unfavourable cognitions and emotions with an optimistic stance. In summation, a considerable cohort of patients manifested a predilection for upholding a sanguine perspective, functioning as a coping mechanism within the theme of emotional considerations (Table 6).

**TABLE 6:** Articles on the theme of emotional factors.

Author(s)/ Year	Title	Objective(s)	Main Finding(s)
Spendelow et al., 2017 [122]	Coping and adjustment in men with prostate cancer: a systematic review of qualitative studies	The current review sought to systematically review and summarise these studies.	A total of five coping strategy categories or ‘metathemes ’ were identified across included studies. These categories were labelled ‘avoidance, minimisation, and withdrawal’, ‘directing cognition and attention’, ‘reframing masculinity and seeking support’, ‘retaining pre‐illness identity and lifestyle’, and ‘symptom/side‐effect management’.
Ghodraty‐Jabloo et al. 2015 [53]	Keep your mind off negative things: Coping with the long‐term effects of acute myeloid leukemia (AML)	The purpose of our study was to explore how AML survivors describe their longer‐term physical and psychosocial well‐being and how they cope with these challenges.	A range of emotion‐ and problem‐focused coping strategies was reported. Keeping one's mind off negative things through engaging in formal work or informal activities and seeking control were the two most commonly used coping strategies. Seeking social support for reassurance was also common. Problem‐focused strategies were frequently described by the ongoing treatment group to manage treatment side effects.
Van der Biessen et al., 2018 [123]	Understanding how coping strategies and quality of life maintain hope in patients deliberating phase 1 trial participation.	This study aimed to understand how hope and motivation of patients considering phase I trial participation are affected by psychological factors such as coping strategies and locus of control (LoC) and general well‐being as measured by the quality of life (QoL).	Patients considering phase I trial participation seem to use a pact of tenacious and flexible coping and control to stay hopeful. Furthermore, hope and QoL positively affected each other. The psychological pact may promote an adaptation enabling them to adjust to difficult circumstances by unconsciously ignoring information, called dissonance reduction.
Religioni et al. 2017 [51]	Strategies of Coping with Pain in Cancer on the Basis of Lung, Breast, Colorectal, and Prostate Carcinoma	The objective of the study was to assess the influence of the primary site and socioeconomic factors on strategies of coping with pain in patients diagnosed with breast, lung, colorectal, and prostate carcinoma.	The strategy the patient selects in order to cope with disease considerably mediates both pain sensation and the quality of patient life
Kenne Sarenmalm et al., 2017 [52]	Relationship of sense of coherence to stressful events, coping strategies, health status, and quality of life in women with breast cancer	To test the hypothesis that Antonovsky's concept of sense of coherence (SOC) predicts stressful events, coping strategies, health status, and quality of life (QoL) in a cohort of postmenopausal women	Sense of coherence significantly predicts distress, number and type of coping strategies such as direct action and relaxation, health status, and QoL in women with breast cancer. Our data suggest that the SOC scale may be a useful screening tool to identify individuals particularly vulnerable to distress and unable to cope adequately.
Lafaye et al., 2014 [86]	Dyadic effects of coping strategies on emotional state and quality of life in postrate cancer patients and their spouses	The aim of this study is to examine the dyadic effects of coping strategies on the emotional state and QoL of couples dealing with cancer.	Results obtained showed that coping strategies used by patients or spouses play a key role not only in their own well‐being but also in their partners’. Indeed, when patients use problem‐focused coping or social support‐seeking, they, as well as their spouses, experience fewer anxiety and depressive symptoms. Conversely, patients or spouses who use emotion‐focused coping experience higher levels of anxiety and depressive symptoms.
Dalnim Cho et al., 2013 [102]	Emotional approach coping: Gender difference on psychological adjustment in young to middle‐aged cancer survivours	We investigated whether the effects of two kinds of Emotional Approach Coping – emotional processing (EP) and emotional expression (EE) – vary by gender and whether EAC has effects above and beyond the effect of other coping strategies.	Gender differences held true in cancer survivors, and EAC was effective when other coping strategies were controlled. Further, EE was effective in reducing negative adjustment in men while EP was helpful in promoting positive adjustment in women.
Ramezanzade Tabriz et al., 2018 [72]	Relationship with unpleasant emotions(anxiety, stress, and depression) and religious coping in pain coping strategy and their cancer patients	To determine the relationship between pain coping and unpleasant emotions as well as religious coping	Patients use prayer as a therapeutic coping strategy to control and cope with their illness
Ichikura et al., 2020 [124]	Patterns of stress coping and depression among patients with head and neck cancer (HNC): A Japanese cross‐ sectional study.	The aim of this study was to (a) identify the clusters of HNC patients based on their stress coping strategies and (b) evaluate the differences in clinical data and depression among the identified HNC patients' coping clusters	This study indicates that patients with a dependent‐coping pattern may account for the largest HNC population and are likely to suffer from depression. Dependent coping includes smoking, drinking, seeking support, or engaging self‐distraction.
Mosher et al., 2015 [55]	Coping with physical and psychological symptoms: a qualitative study of advanced lung cancer patients and their family caregivers	This study aimed to identify strategies for coping with various physical and psychological symptoms among advanced, symptomatic lung cancer patients and their primary family caregivers.	Patients and caregivers reported maintaining a normal routine and turning to family and friends for support with symptom management, which often varied in its effectiveness. Whereas support from health‐care professionals and complementary and alternative medicine were viewed favorably, reactions to Internet and in‐person support groups were mixed due to the tragic nature of participants’ stories. Several cognitive coping strategies were frequently reported (i.e., changing expectations, maintaining positivity, and avoiding illness related thoughts) as well as religious coping strategies.
Lakkis et al., 2015 [125]	Psychological distress and coping strategy in parents of children with cancer in Lebanon	To determine the prevalence of psychological distress (PD) among parents of Lebanese children with cancer and to investigate the associated stressors and coping strategies.	PD is prevalent among parents of Lebanese children hospitalised because of cancer.
Ghiggia et al., 2017 [50]	Psychological distress and coping in nasopharyngeal cancer: an explorative study in Western Europe	The aim of this explorative study is to investigate the psychological distress, coping strategies and quality of life of NPC patients in the post‐treatment observation period.	Results showed that patients with high anxiety or depressive symptoms seem to use dysfunctional coping strategies, such as hopelessness and anxious preoccupation, more than patients with lower levels of anxiety and depression
Pahlevan Sharif et al., 2018 [87]	Religious coping and death depression in Iranian patients with cancer: relationships to disease stage	The study investigated relationships among the extent of disease, religious coping, and death depression in Iranian patients with cancer.	After controlling for demographic and health characteristics, positive and negative religious coping behaviors were significantly related to the experience of death depression. There was an interaction effect between negative religious coping and extent of disease with significant positive relationships to the experience of death depression.
Roberts et al., 2018 [41]	A revised model for coping with advanced cancer. Mapping concepts from a longitudinal qualitative study of patients and carers coping with advanced cancer onto Folkman and Greer's theoretical model of appraisal and coping	To explore whether the Folkman and Greer theoretical model of appraisal and coping reflects the processes used by people living with advanced cancer	This study revealed that people with advanced cancer develop ways of “living around” and “living with” that which comes with the cancer. The coping process for people with advanced cancer appears non‐linear, fluctuating between positive and negative issues additional events, threats, harms, and challenges.
Schreiber J.A. 2011 [88]	Image of God: Effect on coping and psycho‐spiritual outcomes in early breast cancer survivours	To examine the effect of breast cancer survivors’ views of God on religious coping strategies, depression, anxiety, stress, concerns about recurrence, and psychological well‐being.	The belief in an engaged God is significantly related to increased psychological well‐being, decreased psychological distressed and decreased concern about recurrence
Jaser et al., 2017 [126]	Coping and psychological distress in mothers of adolescent with type 1 diabetes	The objective of this study was to describe coping in mothers of adolescents with type 1 diabetes and to examine the association among mothers’ diabetes‐related stress and coping strategies and maternal psychological distress and diabetes‐related family conflict, and glycemic control.	Maternal stress and depressive symptoms are linked with negative outcomes in adolescents, including deteriorating glycemic control, poorer quality of life, and greater depressive symptoms
Yasui‐Furukori et al., 2019 [43]	Coping behaviours and depressive status in individuals with type 2 diabetes mellitus	The association between coping behaviours and the reported levels of depressive symptoms	Avoidance and suppression, emotional expression involving others and changing one's point of view were significantly associated with depressive symptoms. Therefore, coping behaviours may affect depressive symptoms among T2DM.
Arnold et al., 2016 [68]	Coping with the economic burden of Diabetes, TB and co‐prevalence: Evidence from Bishkek, Kyrgyzstan	This study aims to explore how households cope with the economic burden of Diabetes, TB and co‐prevalence.	This study shows that while OOP is moderate for TB‐affected patients, there are severe consequences for Diabetes affected patients. As a result of the underfunding of the SGBP, Diabetes and co‐affected patients are challenged by OOP. Especially those who belong to lower socio‐economic groups are challenged in coping with the economic burden.
Rassart et al., 2016 [105]	Coping with type 1 diabetes through emerging adulthood: Longitudinal association with perceived control and hemoglobin A1c	To examine how perceived personal control, coping, and HbA_1c_ relate to one another over time.	Over the course of the study, patients showed a worsening of glycaemic control and a decrease in perceived personal control. A longer illness duration has indeed been associated with poorer glycaemic control, more severe hypoglycaemic events, and poorer dietary self‐care in individuals with type 1 diabetes.
Bazzazian S. and Besharat M.A. 2012 [48]	An exploratory model of adjustment to type 1 diabetes based on attachment, coping, and self‐regulation theories	This study aimed to develop and test a model of adjustment to type I diabetes.	Positive illness perception and more usage of task‐oriented coping strategy predict better adjustment to diabetes. So, the results confirmed the theoretical bases and empirical evidence of effectiveness of attachment styles in adjustment to chronic disease and can be helpful in devising preventive policies, determining high‐risk maladjusted patients, and planning special psychological treatment.
Hart, P.L. and Gatson, Grindel, C. 2010 [73]	Illness representations, emotional distress, coping strategies, and coping efficacy as predictors of patients' outcomes in type 2 diabetes	To understand the degree of influence that psychosocial factors have on individuals’ decisions to perform self‐regulation activities	Participants perceived their diabetes to be a chronic, moderately cyclical condition with negative consequences and with moderate amounts of symptomatology that greatly influenced their emotional status.
Şahin et al., 2015 [127]	Assessment of psychopathology, quality of life, and parental attitudes in adolescents with Type 1 Diabetes Mellitus	The present study aimed to identify psychopathology, parental attitudes, perceptions of quality of life, and relationships between these factors in adolescents with type 1 diabetes mellitus (DM).	A high rate of psychopathology was detected among adolescents with type 1 DM. In addition, no clear impairment in quality of life was reported in patients with type 1 DM; however, there was worsening in the perception of quality of life in the presence of psychiatric disorders accompanying diabetes. It was found that parents of diabetic children use inappropriate coping strategies and negative parental attitudes more often than those of healthy controls.
Jaser et al. 2012 [128]	Coping and psychological distress in mothers of adolescents with type 1 diabetes	The objective of this study was to describe coping in mothers of adolescents with type 1 diabetes and to examine the association among mothers’ diabetes‐related stress and coping strategies and maternal psychological distress and diabetes‐related family conflict, and glycemic control.	Maternal stress and depressive symptoms are linked with negative outcomes in adolescents, including deteriorating glycemic control, poorer quality of life, and greater depressive symptoms.
Brassington et al., 2016 [101]	Better living with illness: A trans diagnostic acceptance and commitment therapy group intervention for chronic physical illness	To assess how commonalities across long‐term conditions will be beneficial for people with long‐term health conditions	Improvements were seen in depression and anxiety symptoms, limitations of the health condition, and valued living. Health status and health appraisals did not change.
Konijzer et al., 2018 [129]	COPD‐related fatigue: Impact on daily life and treatment opportunities from the patient's perspective	This study addresses the patient's perspective on the impact of fatigue on their daily lives and explores possible treatment options to tackle the burden of fatigue.	Patients perceived a severe negative impact of fatigue on their daily lives and emphasized that they were limited in physical, emotional, cognitive, and social functioning.
Petticrew et al., 2014 [130]	Influence of psychological coping on survival and recurrence in people with cancer: Systematic review	To summarise the evidence on the effect of psychological coping styles (including fighting spirit, helplessness/hopelessness, denial, and avoidance) on survival and recurrence in patients with cancer	There is little evidence that psychological coping styles play an important part in survival from or recurrence of cancer. People with cancer should not feel pressured into adopting particular coping styles to improve survival or reduce the risk of recurrence.

### 3.10 Stress Coping (Maladaptive) Strategies

The theme of maladaptive strategies was included in fourteen (14) articles in this review. ‘Maladaptive behaviour’ refers to actions that impede an individual's capacity to effectively adapt to situations. Prevalent among individuals with social anxiety disorder (SAD), these behaviours are frequently employed to momentarily alleviate anxiety and fear. Maladaptive strategies of coping with chronic disease happen to individuals who slip into hopelessness and cling to substance abuse out of social exclusion. These psychological problems are common denominators for patients with chronic disease, accounting for 7%–50% of all such patients [71, 73, 100, 104].

Incidentally, the rate of suicide among patients is four times higher than any other patient [100]. Moreover, depression is a conduit for low QoL, poor mental resilience with high propensity to pain, poor adherence to treatment and poor survival rate [41, 48, 55, 69, 70, 72, 92, 100, 104]. Cancer is regarded as incurable, and patients reported disruptions to the fabric of ‘number life’.

I was just shocked that after 20 years, the breast cancer is back …. Took me a while to accept it …. Live with it basically—female participants. [41]

### 3.11 External Strategies

#### 3.11.1 Expanding Social Worlds

The second organising theme from the review deals with the patients’ interaction with close people around them in seeking to expand their social world. Fifteen (15) out of the fifty‐four (54) articles mentioned this theme. Social aspects of coping programmes were also critical for participants as they gave them important benefits from interaction with close people around them [43, 46, 56, 101, 44]. Expanding social worlds has been defined variously, but Thoits [81] defines it to include the extent to which a person's need for affection, approval, belonging and security is met by significant others (SO). Yet, it is widely acknowledged that the spouse is usually the first source of support, though others can also support, whereas children tend to depend more on support from parents. This article supports earlier works that emphasised the helpfulness of social support in improving adjustment and disease symptoms, which brings about higher self‐esteem [106, 107]. The social world of people with chronic conditions is twofold, namely, healthcare providers and family or support groups. In one of the studies, a participant who was living with diabetes succinctly captured the friendship relationship as follows:

It's like sitting down with my sister or neighbour … she'd come in, take her coat off, sit down, get a cup of tea … she was a natural. [68]

Chronic disease patients pinpointed that social contact with other chronic patients was a key component of group‐based self‐management programmes, which improves psychological wellbeing. This group of people suffers from social isolation because the disease limits their physical functions, and the illness condition usually places a social stigma on them as society perceives past smoking behaviour to be self‐inflicted [43, 45, 46, 69, 42, 86, 108, 109, 110]. As a result, peer support was valuable as it provided them with the unique experiences of empathising with one another. A complementary part was the group aspect of the exercise programme that fits well in this theme and not only provides social support but is considered by the patients as motivating, inspiring and stimulating [43, 46, 70, 108]. A study by Baker and Fatoye [110] based on an online self‐management diary found that participants expressed a desire to have at least a social fellowship that ensures fellow feeling as they strive to do physical activity. Some participants contrasted their exercises vis‐à‐vis that with their peers as not interesting. One patient reported that

I think I need others to get me up. I do the ones that don't bore me, but not every day. [34]

Although this study was an internet‐based self‐management programme, it was found that this self‐management programme was effective in improving long‐term conditions, including diabetes [111]. However, a recent Cochrane review was unable to ascertain the efficacy of these interventions for individuals with chronic diseases, primarily due to the limited availability of high‐quality studies on which to ground definitive recommendations [113]. The prevalence of extensive social networks alongside limited levels of health literacy potentially diminishes the effectiveness of computer‐based self‐management interventions as a means of furnishing support for individuals grappling with these chronic diseases. Unquestionably, the literature examined underscores the pivotal role played by healthcare providers as significant social actors. In this way, they provide support that enhances psychological well‐being together with their clinical and medical focus [70, 80].

Conventionally, the foundation of social support rests upon familial members, spouses, intimate companions, caregivers and support groups characterised by shared experiences [42, 46, 69, 76, 112]. Expanding social worlds therefore play varied roles, as, for instance, support groups provide patient with various information which makes them better understand their symptoms, so they can better cope with the symptoms and empathise with each other as they are all united by a common denominator—chronic disease. It seems that the social world, when active in the life of a patient, brings about a critical partnership that incubates behavioural change through information and strategy dissemination and builds the capacity of the patient to make informed choices to better manage these chronic conditions (Table 7).

**TABLE 7:** Articles on the theme of expanding social worlds.

Author(s)/ Year	Title	Objective(s)	Main Finding(s)
Jibb et al., 2018 [49]	Children's experiences of cancer care: A systematic review and thematic synthesis of qualitative studies	The aim was to systematically review, appraise, and synthesize the evidence describing the life experiences (physical, emotional, cognitive, and social) of children receiving cancer treatment.	Children and adolescents are profoundly affected by cancer treatment. Young patients find themselves and their families changed, live in a juxtaposition between changing realities, are physically and psychosocially burdened by therapy, face stigmatisation, and long for normalcy, but can also develop resilience.
Bennett et al., 2012 [84]	Concerns and coping during cancer genetic risk assessment.	To gain an ‘in‐depth’ understanding of patients’ concerns and their related coping strategies during the genetic risk assessment process.	The most highly endorsed coping strategies were emotion‐focused: positive appraisal, acceptance, and seeking support from family or friends.
Ghodraty‐Jabloo et al., 2015 [53]	Keep your mind off negative things: Coping with the long‐term effects of acute myeloid leukemia (AML)	The purpose of our study was to explore how AML survivors describe their longer‐term physical and psychosocial well‐being and how they cope with these challenges.	A range of emotion‐ and problem‐focused coping strategies was reported. Keeping one's mind off negative things through engaging in formal work or informal activities and seeking control were the two most commonly used coping strategies. Seeking social support for reassurance was also common. Problem‐focused strategies were frequently described by the ongoing treatment group to manage treatment side effects.
Kenne Sarenmalm et al., 2017 [52]	Relationship of sense of coherence to stressful events, coping strategies, health status, and quality of life in women with breast cancer	To test the hypothesis that Antonovsky's concept of sense of coherence (SOC) predicts stressful events, coping strategies, health status, and quality of life (QoL) in a cohort of postmenopausal women	Sense of coherence significantly predicts distress, number and type of coping strategies such as direct action and relaxation, health status, and QoL in women with breast cancer. Our data suggest that the SOC scale may be a useful screening tool to identify individuals particularly vulnerable to distress and unable to cope adequately.
Lafaye et al., 2014 [86]	Dyadic effects of coping strategies on emotional state and quality of life in postrate cancer patients and their spouses	The aim of this study is to examine the dyadic effects of coping strategies on the emotional state and QoL of couples dealing with cancer.	Results obtained showed that coping strategies used by patients or spouses play a key role not only in their own well‐being but also in their partners’. Indeed, when patients use problem‐focused coping or social support‐seeking, they, as well as their spouses, experience fewer anxiety and depressive symptoms. Conversely, patients or spouses who use emotion‐focused coping experience higher levels of anxiety and depressive symptoms.
Vikki Knott et al., 2012 [46]	A grounded theory approach to understand the cancer‐ coping process	The aim of this study was to conduct an inquiry, which focused on the ‘lived experience’ and the social context within which communication about cancer occurs.	A research focus on the social environment in which cancer is experienced provides considerable insight into the cancer‐coping process especially through communication.
Ichikura et al., 2020 [124]	Patterns of stress coping and depression among patients with head and neck cancer (HNC): A Japanese cross‐ sectional study.	The aim of this study was to (a) identify the clusters of HNC patients based on their stress coping strategies and (b) evaluate the differences in clinical data and depression among the identified HNC patients' coping clusters	This study indicates that patients with a dependent‐coping pattern may account for the largest HNC population and are likely to suffer from depression. Dependent coping includes smoking, drinking, seeking support, or engaging self‐distraction.
Scrignaro et al., 2011 [42]	The combined contribution of social support and coping strategies in predicting post‐traumatic growth: a longitudinal study on cancer patients	The aim of this study is to investigate the role of social support and coping strategies in enhancing post‐traumatic growth (PTG) in cancer patients.	Cancer patients who received support from family and friends that satisfied their psychological needs experienced more post –traumatic growth (PTG) in the short term. A greater attempt to engage with and manage stressors and problems associated with the cancer experience was crucial.
Yasui‐ Furukori et al., 2019 [43]	Coping behaviours and depressive status in individuals with type 2 diabetes mellitus	The association between coping behaviours and the reported levels of depressive symptoms	Avoidance and suppression, emotional expression involving others and changing one's point of view were significantly associated with depressive symptoms. Therefore, coping behaviours may affect depressive symptoms among T2DM.
Lawson et al., 2010 [131]	Mediation by illness perceptions of the effect of personality and health threat communication on coping with the diagnosis of diabetes	To examine relationship of personality traits and diabetes health to communication and to coping strategies of newly diagnosed diabetes patients	Both personality traits and health threat communication predict the way individuals cope with disease
Koerich et al., 2013 [47]	Myocardial revascularization: Strategies for coping with the disease and the surgical process	The study aimed to know the strategies used by patients in coping with coronary heart disease and the surgical process of myocardial revascularization.	The main strategies used by patients to cope with coronary cardiac disease and the process are the presence and support of the family, the quality of interfamily relationships, the use of spiritual resources and participation in rehabilitation programmes that in addition to physical fitness, enable social interactions.
Kouijzer et al., 2018 [108]	COPD‐related fatigue: Impact on daily life and treatment opportunities from the patient's perspective	This study addresses the patient's perspective on the impact of fatigue on their daily lives and explores possible treatment options to tackle the burden of fatigue.	Patients perceived severe negative impact of fatigue on their daily lives and emphasized that they were limited in physical, emotional, cognitive and social functioning.
Yadav et al., 2018 [44]	Self‐ management and patient's activation in COPD patients. An evidenced summary of randomized controlled trials	To identify the benefits and knowledge gaps in self‐management and patient‐activation position in COPD self‐management interventions	Within a heterogeneity of self‐management interventions, it is difficult to determine the most effective among them for there is no uniformity of consent and mode of coping strategy except perhaps the one the individual identifies most with.
Chatrung et al., 2015 [54]	Wellness and religious coping among Thai individuals living with chronic kidney disease (CKD) in Southern California.	To assist healthcare professionals in developing and implementing educative and supportive interventions for patients with CKD.	The results showed an interrelationships of four (4) emerging categories to have a direct impact on chronic disease‐ family relations, social support, knowledge/awareness and religious coping affect self‐care and how one manages one's life.
Mosher et al., 2015 [55]	Coping with physical and psychological symptoms: a qualitative study of advanced lung cancer patients and their family caregivers	This study aimed to identify strategies for coping with various physical and psychological symptoms among advanced, symptomatic lung cancer patients and their primary family caregivers.	Patients and caregivers reported maintaining a normal routine and turning to family and friends for support with symptom management, which often varied in its effectiveness. Whereas support from health‐care professionals and complementary and alternative medicine were viewed favorably, reactions to Internet and in‐person support groups were mixed due to the tragic nature of participants’ stories. Several cognitive coping strategies were frequently reported (i.e., changing expectations, maintaining positivity, and avoiding illness‐related thoughts) as well as religious coping strategies.

### 3.12 CTs and Alternative Medicine—CAM Use

**FIGURE 7:**
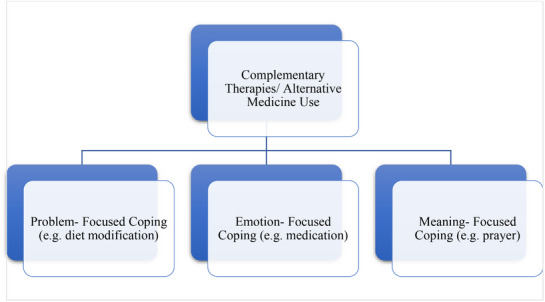
Sub‐themes under complementary therapies and alternative medicine use under external coping strategies.

Under the CTs theme, as many as fifteen (15) studies included in the current review featured this theme (Table 8). The use of CTs as an extra strategy is increasing in almost every part of the world, particularly in Australia [77, 85]. Two distinct categories of CTs exist: first, those involving the utilisation of natural and biologically derived substances (including dietary supplements, herbal remedies and specialised diets); and second, modalities encompassing mind‐body practices such as acupressure/acupuncture, exercise/movement therapies, massage, meditation/relaxation, spiritual engagements and yoga [78]. Yun et al. [79] asserted that CTs do not increase survival rates, yet compelling evidence underscores their applicability within the context of supportive cancer therapy. Notably, practices like acupuncture and exercise demonstrate potential in ameliorating disease symptoms and treatment‐related adverse effects. Furthermore, interventions such as meditation, yoga, aromatherapy and exercise contribute substantively to the well‐being and overall QoL for patients [77]. Another Australian longitudinal study reported that there are long‐term benefits of CTs in women diagnosed with cancer [114]. CTs played a pivotal role in mitigating stress among cancer patients. A recurrent thematic pattern emerged, indicating that CTs notably facilitated coping among male individuals grappling with an array of physical, psychological and socio‐spiritual symptoms. This coping mechanism exhibited three primary branches: problem‐, emotion‐ and meaning‐focused coping (Figure 7).

**TABLE 8:** Articles on the theme of stress (maladaptive) coping strategies.

Author(s)/ Year	Title	Objective(s)	Main Finding(s)
Ichikura et al., 2020 [124]	Patterns of stress coping and depression among patients with head and neck cancer. A Japanese cross‐sectional study	This study aimed to (a) identify the clusters of HNC patients based on their stress coping strategies and (b) evaluate the differences in clinical data and depression among the identified HNC patients' coping clusters.	This study indicates that patients with a dependent‐coping pattern may account for the largest HNC population and are likely to suffer from depression. Dependent coping includes smoking, drinking, seeking support, or engaging in self‐distraction.
Ghiggia et al., 2017 [50]	Psychological distress and coping in nasopharyngeal cancer: an explorative study in Western Europe.	The aim of this explorative study is to investigate the psychological distress, coping strategies and quality of life of NPC patients in the post‐treatment observation period.	Results showed that patients with high anxiety or depressive symptoms seem to use dysfunctional coping strategies, such as hopelessness and anxious preoccupation, more than patients with lower levels of anxiety and depression.
Roberts et al., 2018 [41]	A revised model for coping with advanced cancer. Mapping concepts from a longitudinal qualitative study of patients and carers coping with advanced cancer onto Folkman and Greer's theoretical model of appraisal and coping	To explore whether the Folkman and Greer theoretical model of appraisal and coping reflects the processes used by people living with advanced cancer	This study revealed that people with advanced cancer develop ways of “living around” and “living with” that which comes with the cancer. The coping process for people with advanced cancer appears non‐linear, fluctuating between positive and negative issues, additional events, threats, harms, and challenges.
Yu et al., 2011 [132]	Coping mediates the association between Type D personality and perceived health in Chinese patients with coronary heart disease (CHD).	This study examined the association between Type D personality, coping, and perceived health among Chinese patients with coronary heart disease (CHD).	The Type D patients used maladaptive coping in response to the disease. These coping strategies fully mediated the association between Type D personality and perceived health. They tend to be vulnerable to the low perception of health status, while they employ acceptance–resignation, which in turn makes these patients suffer from low morale.
Mosher et al., 2015 [55].	Coping with physical and psychological symptoms: A qualitative study of advanced lung cancer patients and their family care givers.	To identify strategies for coping with various physical and psychological symptoms.	Results suggest that advanced lung cancer patients and caregivers may be more receptive to cognitive and religious approaches to symptom management and less receptive to peer support. Intervention should address the perceived effectiveness of support from family and friends.

### 3.13 CTs Are Used as Problem‐Focused Coping

Within this sub‐theme, the utilisation of natural and biologically derived products is undertaken with the intent of enhancing the body's condition during cancer treatment. This includes endeavours to optimise the impact of chemotherapy or radiotherapy, aiming for maximal efficacy. As an illustration, an individual afflicted with non‐metastatic lung cancer articulated the following sentiment:

Food supplements, herbs, various herbal supplements … you think they're gonna help cure your cancer.… You feel that there's some hope in there … you feel that that can boost your immune system, and also that some of those will have a cancer‐killing effect … (I) felt better … had some sort of control over … treatment and … outcomes. [85]

Besides this, prayer routines alleviate anxious, uncertain and depressed situations [85]. Offering individuals in such circumstances a foundation of tranquillity, direction and interpersonal bonds to bolster coping mechanisms rooted in deriving meaning over an extended duration [47, 50, 55, 85, 115]. These rationales underscore the physiological bases for the utilisation of CTs as a problem‐focused response [47, 48, 70, 72, 41, 73, 85, 100]; men also enhance their physiological well‐being and bolster their immune systems through the consumption of natural products. Cancer patients expressed that the utilisation of CTs contributes to prolonged survival. In contrast, males diagnosed with non‐metastatic cancer, implying a potential for cure, consistently indicated their utilisation of these CTs as a preventative measure against cancer recurrence. These instances underscore the significance of their conviction in the efficacy of these approaches for averting cancer relapse [101]. These statements about prophylaxis against relapse and extension of survival represent a distinct emotion‐focused coping strategy, particularly when viewed from a physician's perspective [116], further supporting the axiom that using CTs reduces anxiety. In the reviewed articles, patients and their SO said that nature and biologically based CTs, when consumed, improve their physical outcomes. This, from the patient's perspective, points to the problem‐focused strategy. It is also possible for CTs to fulfil multiple coping strategies, especially from the perspective of different standpoints—patient, SO, physician and researchers. Illustratively, herbal medicines might be consumed as a means of prevention with a problem‐focused prevention [113]; however, their consistent and systematic utilisation could also contribute to anxiety reduction, so that this CT use could also be seen as emotion‐focused coping [69, 113]. Similarly, yoga functions as a method to alleviate distress and enhance overall well‐being, aligning with the framework of emotion‐focused coping. Finally, the use of prayer helps patients to cope with uncertainties, anxieties and feelings of depression. Spiritual practices generally help patients with chronic conditions enjoy peace, purpose and interpersonal connection. One can also say prayer is a meaning‐based coping strategy [85].

### 3.14 CTs Are Used for Emotion‐Focused Coping

Another distinctive sub‐theme within the realm of CTs pertains to emotion‐focused coping. This facet of coping proves valuable in addressing psychologically distressing circumstances, such as instances marked by uncertainty, anxiety and depression. For example, one man with brain cancer stated that

(Yoga/meditation) It is a way of stretching the body and the mind and the soul … disease has been a big burden, but with Yoga, you can let go, and that's a big help. [85]

Established CT users do so to maintain a regular balance of mind and body, but some have difficulty getting this result. Men's SO did not always contribute to men's decision to take up or maintain mind and body practices. This is often the case because coping with emotions is a matter of privacy [85]. A spouse characterised her husband's consistent engagement in yoga and meditation as a personal undertaking.

He determines that himself, he doesn't do it every day … I know he needs that space sometimes, that might allow me to go into the garden, and do the same for myself. [85]

SO concurred with the aforementioned observations, acknowledging the necessity for patients to independently manage distressing emotions [85]. In all, participants generally gained feelings of security, confidence and protection when they saw some physical changes through their exercises.

### 3.15 CTs Are Used for Meaning‐Focused Coping

Another distinct sub‐theme within the domain of CTs pertains to meaning‐focused coping. In this context, a substantial number of men acknowledged the advantages garnered through their customary engagement in CTs, specifically encompassing spiritual practices and prayer [47, 50, 55, 80, 42, 84, 86]. These specific CTs serve to provide solace, offering them a means to grapple with existential inquiries that individuals are confronted with upon diagnosis of life‐threatening ailments [115]. A patient's account further illustrates this sentiment:

When I first got this illness … I asked myself what it all means. What was it all about? …. Now I'm more focused on being open to God, open to the power of what he can do in me, and expecting him to do something in me … both my wife and I have been on the same focus, we both looked at this situation from the same point of view, a very powerful experience. [85]

According to reports, a portion of men maintained a consistent practice of prayer, adhering to specific patterns such as designated times during the morning and evening [85]. Furthermore, others employed prayer as a means to navigate through specific challenging circumstances encountered along their cancer journey. For instance, an individual diagnosed with metastatic lung cancer recounted his utilisation of prayer during instances when he experienced particular feelings (Table 9).

Bad, really crooked, really sick … then I spend time praying about it and it does relieve the tension, it gives you confidence, it gives you peace of mind … so you can overcome that feeling of being so sick. [85]

In one study, an individual conveyed that he turned to prayer during periods when he experienced a sense of being ‘fairly ordinary’, attributing prayer's role in inducing relaxation and facilitating restful sleep. Additionally, men, alongside their SO, occasionally engaged in joint prayer and participated in meaning‐focused coping strategies as indicated by a spouse's statement [85].

Prayer … would have to be our priority, more so than any other thing is prayer, to both of us …. We are very motivated in exploring the depth of prayer, the depth of spirituality …. We pray together, we have a regime of prayer, every day. [85]

There are times prayer becomes a shared responsibility with patients and their SO more particularly when they share the same spiritual community. The support of friends and like‐minded people joining in collective prayer alongside and on behalf of patients brings positive thoughts and experiences that improve physical states. For instance, a patient diagnosed with metastatic colorectal cancer articulated the following perspective:

By praying alone and praying together, I think I felt emotionally better, and not just mentally. Physically follows on from that, I think if you're emotionally feeling okay, and mentally you're feeling okay, then physically you feel better. [85]

**TABLE 9:** Articles on the theme of complementary therapies.

Author(s)/ Year	Title	Objective(s)	Main Finding(s)
Spendelow et al., 2017 [122]	Coping and adjustment in men with prostate cancer: a systematic review of qualitative studies	The current review sought to systematically review and summarise these studies.	A total of five coping strategy categories or ‘metathemes ’ were identified across included studies. These categories were labelled ‘avoidance, minimisation, and withdrawal’, ‘directing cognition and attention’, ‘reframing masculinity and seeking support’, ‘retaining pre‐illness identity and lifestyle’, and ‘symptom/side‐effect management’.
Van der Biessen et al., 2018 [123]	Understanding how coping strategies and quality of life maintain hope in patients deliberating phase of 1 trial participation.	This study aimed to understand how the hope and motivation of patients considering phase I trial participation are affected by psychological factors such as coping strategies and locus of control (LoC) and general well‐being as measured by the quality of life (QoL).	Patients considering phase I trial participation seem to use a pact of tenacious and flexible coping and control to stay hopeful. Furthermore, hope and QoL positively affected each other. The psychological pact may promote an adaptation enabling them to adjust to difficult circumstances by unconsciously ignoring information, called dissonance reduction.
Gaston‐Johansson et al., 2013 [45]	This review is an attempt to identify the benefits and knowledge gaps in self‐management and patient‐activation position in COPD self‐management interventions	This study aims to examine the effectiveness of a self‐management multimodal comprehensive coping strategy program (CCSP) on quality of life (QOL) among breast cancer patients 1 year after treatment	The CCSP improved QOL for patients at 1‐year follow‐up. Patients overwhelmingly reported that CCSP was beneficial. The CCSP as an effective coping intervention has potential as a self‐management program for breast cancer survivors.
Robertson et al., 2018 [41]	A revised model for coping with advanced cancer. Mapping concepts from a longitudinal qualitative study of patients and carers coping with advanced cancer onto Folkman and Greer's theoretical model of appraisal and coping	To explore whether the Folkman and Greer theoretical model of appraisal and coping reflects the processes used by people living with advanced cancer	This study revealed that people with advanced cancer develop ways of “living around” and “living with” that which comes with the cancer. The coping process for people with advanced cancer appears non‐linear, fluctuating between positive and negative issues additional events, threats, harms, and challenges.
Préau et al., 2013 [83]	Two years after cancer diagnosis, what is the relationship between health‐related quality of life, coping strategy and spirituality?	This study aimed to analyse the relationship between spirituality, coping strategies and health‐related quality of life (HRQL) among a large representative sample of patients two years after cancer diagnosis.	The results of this study highlight how spirituality may be a valuable source of comfort during cancer treatment and consequently may play an important role in the management of one's disease. It especially questions about the role of religious actors in providing the support that patients expect as well as helping them to cope with the disease.
Bazzazian S. and Besharat M. A. 2012 [48]	An exploratory model of adjustment to type 1 diabetes based on attachment, coping and self‐ regulation theories.	The aim of this study was to develop and test a model of adjustment to type I diabetes.	Positive illness perception and more usage of task‐oriented coping strategy predict better adjustment to diabetes. So, the results confirmed the theoretical bases and empirical evidence of effectiveness of attachment styles in adjustment to chronic disease and can be helpful in devising preventive policies, determining high‐risk maladjusted patients, and planning special psychological treatment.
Lawson et al., 2010 [131]	Mediation by illness perceptions of the effect of personality and health threat communication on coping with the diagnosis of diabetes	To examine relationship of personality traits and diabetes health to communication and to coping strategies of newly diagnosed diabetes patients	Both personality traits and health threat communication predict the way individuals cope with disease.
Koerich et al., 2013 [47]	Myocardial revascularization: Strategies for coping with the disease and the surgical process	The study aimed to know the strategies used by patients in coping with coronary heart disease and the surgical process of myocardial revascularization.	The main strategies used by patients to cope with coronary cardiac disease and the process are the presence and support of the family, the quality of interfamily relationships, the use of spiritual resources and participation in rehabilitation programmes that in addition to physical fitness, enable social interactions.
Chatrung et al., 2015 [54]	Wellness and religious coping among Thai individuals living with chronic kidney disease (CKD) in Southern California.	To assist healthcare professionals in developing and implementing educative and supportive interventions for patients with CKD.	The results showed an interrelationships of four (4) emerging categories to have a direct impact on chronic disease‐ family relations, social support, knowledge/awareness and religious coping affect self‐care and how one manages one's life.
Nadja et al., 2014 [133]	Australian men with cancer practice complementary therapies as a coping strategy	The aim of this study was to explore how and why Australian men with cancer practice complementary therapies (CTs) and how their significant others (SOs) contribute to the regular uptake of CTs.	Three core themes were identified: men used CTs as (a) problem‐focused coping (e.g., diet modification), (b) emotion‐focused coping (e.g., meditation), and (c) meaning‐based coping (e.g., prayer). Practicing CTs helped men to cope with physical, emotional, and spiritual concerns, although some men spoke of difficulties with practicing meditation to regulate their emotions. SOs were supportive of men's coping strategies but were only rarely involved in men's emotion‐focused coping.
Scrignaro et al., 2011 [42]	The combined contribution of social support and coping strategies in predicting post‐traumatic growth: a longitudinal study on cancer patients	The aim of this study is to investigate the role of social support and coping strategies in enhancing post‐traumatic growth (PTG) in cancer patients.	Cancer patients who received support from family and friends that satisfied their psychological needs experienced more post –traumatic growth (PTG) in the short term. A greater attempt to engage with and manage stressors and problems associated with the cancer experience was crucial.
Kanne Sarenmalm et al., 2017 [52]	Relationship of sense of coherence to stressful events, coping strategies, health status, and quality of life in women with breast cancer	To test the hypothesis that Antonovsky's concept of sense of coherence (SOC) predicts stressful events, coping strategies, health status, and quality of life (QoL) in a cohort of postmenopausal women	Sense of coherence significantly predicts distress, number and type of coping strategies such as direct action and relaxation, health status, and QoL in women with breast cancer. Our data suggest that the SOC scale may be a useful screening tool to identify individuals particularly vulnerable to distress and unable to cope adequately.
Lafaye et al., 2014 [86]	Dyadic effects of coping strategies on emotional state and quality of life in postrate cancer patients and their spouses	The aim of this study is to examine the dyadic effects of coping strategies on the emotional state and QoL of couples dealing with cancer.	Results obtained showed that coping strategies used by patients or spouses play a key role not only in their own well‐being but also in their partners’. Indeed, when patients use problem‐focused coping or social support‐seeking, they, as well as their spouses, experience fewer anxiety and depressive symptoms. Conversely, patients or spouses who use emotion‐focused coping experience higher levels of anxiety and depressive symptoms.
Barsky‐Reese et al., 2010 [100]	Coping with sexual concerns after cancer: the use of flexible coping	To describe a broader approach to understanding and treating sexual concerns in cancer that focuses on the construct of flexibility in behavioural and cognitive coping strategies	We argue that coping flexibly with sexual concerns is likely to lead to improvements in mood and sexual and relationship satisfaction.
Mosher et al., 2015 [55]	Coping with physical and psychological symptoms: a qualitative study of advanced lung cancer patients and their family caregivers	This study aimed to identify strategies for coping with various physical and psychological symptoms among advanced, symptomatic lung cancer patients and their primary family caregivers.	Patients and caregivers reported maintaining a normal routine and turning to family and friends for support with symptom management, which often varied in its effectiveness. Whereas support from health‐care professionals and complementary and alternative medicine was viewed favorably, reactions to Internet and in‐person support groups were mixed due to the tragic nature of participants’ stories. Several cognitive coping strategies were frequently reported (i.e., changing expectations, maintaining positivity, and avoiding illness‐related thoughts) as well as religious coping strategies.

### 3.16 Therapeutic Interventions

Under this theme of therapeutic interventions, a total of seventeen (17) articles were reviewed, and this is shown in Table 10. Being a positive coping strategy, it is mostly received from others in the form of psychological, practical and medical interventions. Therapeutic interventions help patients to improve their physical functioning and psychological well, as well as knowledge or skills to improve the chronic condition. Usually, the therapeutic measures provide more knowledge on chronic conditions and teach new coping skills and also seek to improve adherence behaviour and improve satisfaction in the work or life of patients. Such measures have been shown to produce more benefits in moods [41, 46, 47, 54, 42, 80, 83, 87, 88, 89] disease acceptance [46, 48, 71, 73, 86] and QoL together with improving the knowledge base of chronic patients [50, 55, 56, 92, 102]. It has been realised that apart from interventions focused on training, skills and rehabilitation, psychological therapeutic interventions influence positive outcomes. Jibb et al. [49] in their study in Canada contend that practical resources to manage cancer are underpinned by social support. Ghodraty‐Jabloo et al. [70] also support this point of seeking social support and further submit, among others, that mental disengagement/distancing and seeking control are examples of coping mechanisms used by cancer patients.

**TABLE 10:** Articles on the theme of Therapeutic intervention.

Author(s)/ Year	Title	Objective(s)	Main Finding(s)
Ghodraty‐Jabloo et al., 2015 [53]	Keep your mind off negative things: coping with long‐term effects of acute myeloid leukemia (AML)	The purpose of our study was to explore how AML survivors describe their longer‐term physical and psychosocial wellbeing and how they cope with these challenges.	A range of emotion‐ and problem‐focused coping strategies were reported. Keeping one's mind off negative things through engaging in formal work or informal activities and seeking control were the two most commonly used coping strategies. Seeking social support for reassurance was also common. Problem‐focused strategies were frequently described by the ongoing treatment group to manage treatment side effects.
Van der Biessen et al., 2018 [123]	Understanding how coping strategies and quality of life maintain hope in patients deliberating phase 1 trial participation.	This study aimed to understand how hope and motivation of patients considering phase I trial participation are affected by psychological factors such as coping strategies and locus of control (LoC) and general well‐being as measured by the quality of life (QoL).	Patients considering phase I trial participation seem to use a pact of tenacious and flexible coping and control to stay hopeful. Furthermore, hope and QoL positively affected each other. The psychological pact may promote an adaptation enabling them to adjust to difficult circumstances by unconsciously ignoring information, called dissonance reduction.
Yadav et al., 2018 [44]	Self‐ management and patient's activation in COPD patients. An evidenced summary of randomized controlled trials	To identify the benefits and knowledge gaps in self‐management and patient‐activation position in COPD self‐management interventions	Within a heterogeneity of self‐management interventions, it is difficult to determine the most effective among them for there is no uniformity of consent and mode of coping strategy except perhaps the one the individual identifies most with.
Barsky Reese et al., 2010 [100]	Coping with sexual concerns after cancer: the use of flexible coping	To describe a broader approach to understanding and treating sexual concerns in cancer that focuses on the construct of flexibility in behavioural and cognitive coping strategies	We argue that coping flexibly with sexual concerns is likely to lead to improvements in mood and sexual and relationship satisfaction.
Dalmin Cho et al., 2013 [102]	Emotional approach coping: Gender difference on psychological adjustment in young to middle‐aged cancer survivors.	We investigated whether the effects of two kinds of Emotional Approach Coping – emotional processing (EP) and emotional expression (EE) – vary by gender and whether EAC has effects above and beyond the effect of other coping strategies.	Gender differences were observed in cancer survivors, and EAC was effective when other coping strategies were controlled. Further, EE was effective in reducing negative adjustment in men, while EP helped promote positive adjustment in women.
Ramezanzade Tabriz et al., 2018 [72]	Relationship with unpleasant emotions(anxiety, stress, and depression) and religious coping in pain coping strategy and their cancer patients	To determine the relationship between pain coping and unpleasant emotions, as well as religious coping	Patients use prayer as a therapeutic coping strategy to control and cope with their illness.
Roberts et al., 2018 [41]	A revised model for coping with advanced cancer. Mapping concepts from a longitudinal qualitative study of patients and carers coping with advanced cancer onto Folkman and Greer's theoretical model of appraisal and coping	To explore whether the Folkman and Greer theoretical model of appraisal and coping reflects the processes used by people living with advanced cancer	This study revealed that people with advanced cancer develop ways of “living around” and “living with” that which comes with the cancer. The coping process for people with advanced cancer appears non‐linear, fluctuating between positive and negative issues, additional events, threats, harms, and challenges.
Jaser et al., 2017 [126]	Coping and psychological distress in mothers of adolescents with type 1 diabetes	The objective of this study was to describe coping in mothers of adolescents with type 1 diabetes and to examine the association among mothers’ diabetes‐related stress and coping strategies and maternal psychological distress and diabetes‐related family conflict, and glycemic control.	Maternal stress and depressive symptoms are linked with negative outcomes in adolescents, including deteriorating glycemic control, poorer quality of life, and greater depressive symptoms.
Bazzazian S. and Besharat M.A. 2012 [48]	An exploratory model of adjustment to type 1 diabetes based on attachment, coping, and self‐regulation theories	This study aimed to develop and test a model of adjustment to type I diabetes.	Positive illness perception and more usage of task‐oriented coping strategy predict better adjustment to diabetes. So, the results confirmed the theoretical bases and empirical evidence of the effectiveness of attachment styles in adjustment to chronic disease and can be helpful in devising preventive policies, determining high‐risk maladjusted patients, and planning special psychological treatment.
Chatrung et al., 2015 [54]	Wellness and religious coping among Thai individuals living with chronic kidney disease (CKD) in Southern California.	To assist healthcare professionals in developing and implementing educational and supportive interventions for patients with CKD.	The results showed an interrelationships of four (4) emerging categories to have a direct impact on chronic disease‐ family relations, social support, knowledge/awareness and religious coping affect self‐care and how one manages one's life.
Lafaye et al., 2014 [86]	Dyadic effects of coping strategies on emotional state and quality of life in prostate cancer patients and their spouses	This study aims to examine the dyadic effects of coping strategies on the emotional state and QoL of couples dealing with cancer.	Results obtained showed that coping strategies used by patients or spouses play a key role not only in their own well‐being but also in their partners’. Indeed, when patients use problem‐focused coping or social support‐seeking, they, as well as their spouses, experience fewer anxiety and depressive symptoms. Conversely, patients or spouses who use emotion‐focused coping experience higher levels of anxiety and depressive symptoms.
Scrignaro et al., 2011 [42]	The combined contribution of social support and coping strategies in predicting post‐traumatic growth: a longitudinal study on cancer patients	The aim of this study is to investigate the role of social support and coping strategies in enhancing post‐traumatic growth (PTG) in cancer patients.	Cancer patients who received support from family and friends that satisfied their psychological needs experienced more post –traumatic growth (PTG) in the short term. A greater attempt to engage with and manage stressors and problems associated with the cancer experience was crucial.
Jibb et al., 2018 [49]	Children's experiences of cancer care: A systematic review and thematic synthesis of qualitative studies	The aim was to systematically review, appraise, and synthesize the evidence describing the life experiences (physical, emotional, cognitive, and social) of children receiving cancer treatment.	Children and adolescents are profoundly affected by cancer treatment. Young patients find themselves and their families changed, live in a juxtaposition between changing realities, are physically and psychosocially burdened by therapy, face stigmatisation, and long for normalcy but can also develop resilience.
Religioni et al., 2017 [51]	Strategies of Coping with Pain in Cancer on the Basis of Lung, Breast, Colorectal, and Prostate Carcinoma	The objective of the study was to assess the influence of the primary site and socioeconomic factors on strategies of coping with pain in patients diagnosed with breast, lung, colorectal, and prostate carcinoma.	The strategy the patient selects to cope with the disease considerably mediates both pain sensation and the quality of patient life.
Ahmadi et al., 2013 [134]	Nature as the most important coping strategy among cancer patients: A Swedish survey	To examine the extent to which the results obtained in a qualitative study among cancer patients in Sweden (Ahmadi, Culture, religion and spirituality in coping:	Nature and the possibilities that being in nature and relating to nature offer cancer patients for coping with their illness should be taken more seriously by health care providers, especially those who deal with the therapy of psychological problems that cancer patients face in different phases, such as diagnosis, treatment, and post‐treatment periods.
Langer Bro et al., 2017 [135]	Kind of blue: A systematic review and meta‐analysis of music interventions in cancer treatment.	To analyze and identify the psychological and physical effects of music interventions in cancer treatment.	Music may be a tool in reducing anxiety, mood, and pain among cancer patients in active treatment.Illness representations by participants are usually governed by family and personal history. Also, migrants make modification in their daily routine while rural non migrants rely on traditional remedies to check the side effects of medications and sometimes in the hope of a cure.
Nyaaba et al., 2019 [91]	Illness representations and coping practices for self‐managing hypertension among sub‐Saharan Africans. A comparative study among Ghanaian migrants and non‐ migrant Ghanaian.	How a person's social and physical context shapes their illness representations of hypertension (HTN) and the coping strategies they develop and adapt to mitigate challenges in self‐managing hypertension.	

In most cases, psychological interventions, which are invariably linked to therapeutic interventions, aim at making patients resilient so that life can be more meaningful. Sensation of pain is a function of psychological factors, and the patient identified six (6) cognition strategies they practiced [92]. These comprise mechanisms including attention diversion (attending to alternative responsibilities), reinterpretation of pain perception (acknowledging pain's impact as less significant), catastrophising (excessive pessimism, envisioning worst‐case scenarios), pain avoidance, engagement in prayer or hope, formulation of coping affirmations (holding the belief in pain management feasibility) and the incorporation of behavioural strategies—amplification of behavioural activities within daily life.

The aforementioned interventions exhibit a close correlation with documented improvements in QoL, underscoring the significance of nature as a pivotal coping mechanism among cancer patients in Sweden. In a quantitative inquiry conducted by Ahmadi and Ahmadi [103], participants diagnosed with cancer were tasked with selecting from a pool of 24 distinct factors perceived to ameliorate their emotional well‐being when experiencing stress, sadness or depression amid or following illness. The findings affirm that a notable 68% of cancer patients within Sweden attested to the markedly positive influence of nature in alleviating emotional distress both during and after the period of illness. Music may be a means of reducing anxiety and pain, and improving mood for patients with cancer, and the most effective genre of music intervention was passive listening [56].

In a distinct investigation conducted by Teisberg et al. [90], a cross‐sectional multisite qualitative study involving semi‐structured interviews was undertaken. This study encompassed a cohort of 55 Ghanaians residing in the Netherlands as well as urban and rural areas of Ghana, all diagnosed with hypertension. The research yielded notable outcomes, including a reduction in depressive symptoms and an enhanced level of self‐management, particularly among those with diabetes. The interviews were administered to the 55 participants with hypertension, and the study's methodology employed thematic analysis for data interpretation. The collected data were categorised into two overarching dialogues voiced by migrants, non‐migrants in urban settings and non‐migrants in rural settings. These discussions were grouped under thematic headings, including diagnosis and treatment, as well as the self‐management of hypertension, encompassing seven fundamental themes.

These essential themes include the process of discovering hypertension, immediate responses to the initial diagnosis and its repercussions, adherence to prescribed medications, compliance with dietary recommendations, adherence to guidelines concerning physical activity, alcohol consumption and smoking, adherence to hypertension treatment regimens and the acquisition of information to self‐manage hypertension.

Notably, within the context of diagnosis and treatment, participants’ reflections upon their discovery of hypertension were categorised as follows within respective subgroups:

Migrants ‘I was asked to go for a general check‐up, and they said that I was diabetic, and I also have it (HTN)’. IDI‐Male‐11

The reaction to the initial diagnosis and consequences by one migrant is as follows:

‘I was not surprised because I had seen it, because of my mother and father, I had seen it before, and I wasn't scared.’ IDI‐Female‐04 ‘ I thought it was not a serious sickness, but when my hand died, I saw that it can kill.’ ID‐Male‐19 ‘I was still smoking at the check‐up …. I was getting up and I fell … this hand got dead so … my wife came and helped me. My child called the ambulance, and it came and picked me to the hospital. From that time till today, I have not missed the medicine … so after I had the stroke’. IDI‐Male‐11

On self‐managing hypertension, some of the participants’ quotes are captured as follows:

A female participant who is a non‐migrant dwelling in an urban area had this to say:

I heard that if you take the doctor's drugs too much, it will destroy your kidney or womb or because you to get a different disease so it is not always I take the medicine … my hands get a little bit stiff and the urine is too much … where will I be urinating like that in town? (Laughing) … If I take some today, I won't take it on the next day for like a week. IDI‐Female‐09

Another female participant, also a non‐migrant in the urban area, had this to say:

Sometimes, too, you cannot get all the medicine, so how will you take it? This disease is for the rich … the government pays some and you have to pay some … the expensive drugs that they write for you to buy outside. IDI‐Female‐10

Aside from the above, some participants’ quotes on adherence to dietary recommendations are captured as follows:

My wife will not cook this rich food for you … I add some magi sauce because I cannot eat … sometimes, I hide and add small salt because all die be die … she always hides the salt (laughing) … she has it [HTN] too, so now the children are not here, she cooks the way the doctor says. IDI‐Male‐08

Before, she cooked separately, or I cooked for myself, but later, you know, cooking is hard, so I let them cook … I hide and eat their food sometimes … You know her children, they will be talking, and you cannot tell them to shut up here (laughing). IDI‐Male‐ 19

Additionally, it became evident that the illness representations and coping strategies for hypertension among participants are influenced by contextual factors within their social and physical surroundings. These influences manifest through dynamic interactions. Several of these contextual elements are exemplified below within the fundamental theme of adhering to dietary recommendations:

Hum, it was not easy. My wife cooks my food separate because of what happened [paralysed hand], but the food is not nice if there is no salt, so I just wait and when I get to the farm, I get my plantain and do something small to eat … she tries but the mouth doesn't like (laughing). IDI‐Male‐08

I cannot say I don't eat salt all the time. When I cook, I don't use salt because my father had it, so I am used to it, so my children eat like that, but you know I trade, so when I am hungry in town, I buy food and eat, and that one has salt. IDI‐Female‐06

### 3.17 Tendency Towards Religion/Music/Natural Romanticism

There is research to support the fact that the physical environment can increase or decrease stress on the human body. What one sees, hears and experiences at any period of time changes one's mood, nervous endocrine and how the immune system works. The stress induced by an unfavourable physical environment can evoke feelings of anxiety, sadness and helplessness. This response can lead to an elevation in blood levels and heart rate, culminating in muscle tension and immune system suppression. Conversely, a conducive physical environment possesses the potential to counteract these effects. Furthermore, irrespective of age or cultural background, humans universally derive pleasure from natural surroundings. In a study documented in the book ‘Healing Gardens’, researchers found that over two‐thirds of individuals opt for natural settings as their refuge in times of stress.

A compilation of 12 articles emerged under the theme encompassing religious, musical and natural coping mechanisms. Numerous patients highlighted their utilisation of church communities, prayer and religious practices to navigate the challenges of chronic conditions, addressing both physical symptoms and emotional responses linked to the illness. The belief in a sovereign and provident God offered comfort and solace, with patients finding reassurance in the idea of divine control over the future. Furthermore, other sources identified that faith and belief in a higher being contribute significantly to coping with the disease. This faith translates into hope and a belief that eventual resolution is attainable. A reflective response encapsulating this sentiment is quoted as follows:

My guardian angel helps me a lot. If we don't hold onto him by our side, light a little candle and say a prayer, we won't make it. [117]

Some studies found that individuals used varying religious beliefs, as well as nature and music, to cope with the stresses that come with their disease. The studies suggested that patients with intrinsic and pro‐religious inclinations cope better with chronic illness [68, 72, 75, 96, 88, 91]. The majority of patients relied on religious practices to navigate their health challenges, engaging in activities such as prayer, meditation, seeking guidance from clergy members and contributing to charitable causes. These were categorised in the sub‐themes below.

**Religious Peace coping**

I always meditate during my dialysis treatment. It helps give me peace and reduces my level of stress. In the past, I was anxious and stressed, but now, I can release stress. I have peace even when I face the possibility of death. [72]

**No miracle**

Principles of religion taught me that everything happens due to my actions. If you want to be healthy, you should adhere to the right diet and fluid restriction, take medication, and stay on your dialysis prescription. [72]

**Accept real life**

‘Religion helps me live with my clinical conditions. When I did meditation or prayer, I wasn't concerned with pain and was able to live with it as a part of my life. After that, I felt at peace and was able to face every problem.’ I do meditation during dialysis, and it helps with deep sleep. [72]

**Religious methods**

‘I have practiced meditation and prayer for more than 10 years. They help me accept the reality of my life.’ I believe in God and that God gives and defines my life. I go to church to pray, and I donate to charity. Giving helps me stay happy. I donate money to the temple and charity. [72]

I had a very, very strong sort of intuitive sense that this illness is …. A spiritual journey, and it has been incredibly wonderful. I almost remember the first day of diagnosis, I could never describe it as anything else but a gift. [78]

The illness made me face up to the fact that I will die someday …. My illness has forced me to understand that I have true spiritual needs, whether I am healthy or unhealthy …. Now, the entire matter of belief is central to me and gives me a truer sense of direction …. These changes, or more accurately, reinforcements, are a precious gift. The cancer gave them to me. I treasure them. [118]

Another article mentioned seeking spiritual closeness with God and natural romanticism as a coping strategy. Over the previous three centuries, Sweden has undergone a transformation towards increased individualism and the development of a secular societal framework. Their studies affirm the romanticisation of nature and closeness with God as two very good coping strategies of people with chronic disease [91]. The faith of these patients’ present health professionals is provided with a subsidy to help them, in that they are able to use these spiritual resources to help patients overcome the difficulties that go with the process of living with myocardial revascularisation surgery [45, 71, 82, 118].

Many patients with chronic disease depend on religion and spirituality to cope in times like this, which affect their QoL. This QoL in context is multidimensional and multifaceted. This health‐related satisfaction has a link with spiritual well‐being, making spirituality and religiosity important factor in coping when one is faced with chronic illness. Religion is a subset of spirituality, even though being religious does not necessarily mean spirituality. Cooper‐Effa et al. [82] posited that spirituality encompasses the capability to transcend life's encounters, embracing life's exuberance and experiencing joy. It is intrinsically linked to the desire for love, acceptance, forgiveness and a sense of fulfilment. Similarly, Vastyan [118] delineated four fundamental constituents crucial to spiritual coping. These encompass the confrontation with the concept of finitude, a state of submission, the occurrence of transcendence (manifesting as a connection to the Divine) and the cherishing of life.

Conversely, religious coping denotes the reliance upon one's religious convictions or rituals to navigate various challenges encountered in life [119]. Within this context, religious coping behaviours encompass activities such as prayer, meditation, participation in religious congregations, engagement with sacred texts or the perception of oneself as religious, all of which influence the approach to managing diverse stressors [136].

Consequently, it is important to provide comprehensive care that addresses the religious, existential and psychosocial requirements of individuals grappling with chronic diseases. Ultimately, the pursuit of peace, well‐being and social support is intrinsic to the fundamental needs of humanity, and it takes on particular significance for those afflicted with chronic illnesses [137].

Other studies suggested music to be an adjunct to cancer treatment as cancer affects all of life's aspects, such as somatic, psychological and social dimensions, thus requiring interdisciplinary approaches to manage patients with chronic diseases [19, 120]. Thus, music can relieve anxiety and pain for patients during cancer treatment. Music soothes the side effects of cancer therapy by reducing anxiety that is associated with chemotherapy and radiotherapy. It can kill nausea and vomiting in patients receiving chemotherapy [138] (Table 11).

**TABLE 11:** Articles on the theme of religious/nature/music coping.

Author(s)/ Year	Title	Objective(s)	Main Finding(s)
Religioni et al., 2017 [51]	Strategies of Coping with Pain in Cancer based on Lung, Breast, Colorectal, and Prostate Carcinoma	The objective of the study was to assess the influence of the primary site and socioeconomic factors on strategies of coping with pain in patients diagnosed with breast, lung, colorectal, and prostate carcinoma.	The strategy the patient selects to cope with the disease considerably mediates both pain sensation and the quality of patient life.
Kenne Sarenmalm et al., 2017 [52]	Relationship of sense of coherence to stressful events, Coping strategies, health status, and quality of life in women with breast cancer	To test the hypothesis that Antonovsky's concept of sense of coherence (SOC) predicts stressful events, coping strategies, health status, and quality of life (QoL) in a cohort of postmenopausal women	Sense of coherence significantly predicts distress, number and type of coping strategies such as direct action and relaxation, health status, and QoL in women with breast cancer. Our data suggest that the SOC scale may be a useful screening tool to identify individuals particularly vulnerable to distress and unable to cope adequately.
Ahmadi et al., 2013 [134]	Nature as the most important coping strategy among cancer patients: A Swedish survey	To examine the extent to which the results obtained in a qualitative study among cancer patients in Sweden (Ahmadi, Culture, religion and spirituality in coping:	Nature and the possibilities that being in nature and relating to nature offer cancer patients for coping with their illness should be taken more seriously by health care providers, especially those who deal with the therapy of psychological problems that cancer patients face in different phases, such as diagnosis, treatment, and post‐treatment periods.
Ramazanzade Tabriz et al., 2018 [72]	Relationship with unpleasant emotions(anxiety, stress, and depression) and religious coping in pain coping strategy and their cancer patients	To determine the relationship between pain coping and unpleasant emotions, as well as religious coping	Patients use prayer as a therapeutic coping strategy to control and cope with their illness.
Mosher et al., 2015 [55]	Coping with physical and psychological symptoms: a qualitative study of advanced lung cancer patients and their family caregivers	This study aimed to identify strategies for coping with various physical and psychological symptoms among advanced, symptomatic lung cancer patients and their primary family caregivers.	Patients and caregivers reported maintaining a normal routine and turning to family and friends for support with symptom management, which often varied in its effectiveness. Whereas support from health‐care professionals and complementary and alternative medicine was viewed favorably, reactions to Internet and in‐person support groups were mixed due to the tragic nature of participants’ stories. Several cognitive coping strategies were frequently reported (i.e., changing expectations, maintaining positivity, and avoiding illness‐related thoughts) as well as religious coping strategies.
Pahlevan–Sharif et al., 2018 [103]	Religious coping and death depression in Iranian patients with cancer: relationships to disease stage	The study investigated relationships among the extent of disease, religious coping, and death depression in Iranian patients with cancer.	After controlling for demographic and health characteristics, positive and negative religious coping behaviors were significantly related to the experience of death depression. There was an interaction effect between negative religious coping and the extent of disease, with significant positive relationships to the experience of death and depression.
Roberts et al., 2018 [41]	A revised model for coping with advanced cancer. Mapping concepts from a longitudinal qualitative study of patients and carers coping with advanced cancer onto Folkman and Greer's theoretical model of appraisal and coping	To explore whether the Folkman and Greer theoretical model of appraisal and coping reflects the processes used by people living with advanced cancer	This study revealed that people with advanced cancer develop ways of “living around” and “living with” that which comes with the cancer. The coping process for people with advanced cancer appears non‐linear, fluctuating between positive and negative issues, additional events, threats, harms, and challenges.
Préau et al., 2013 [83]	Two years after cancer diagnosis, what is the relationship between health‐related quality of life, coping strategy, and spirituality?	This study aimed to analyse the relationship between spirituality, coping strategies, and health‐related quality of life (HRQL) among a large representative sample of patients two years after cancer diagnosis.	The results of this study highlight how spirituality may be a valuable source of comfort during cancer treatment and consequently may play an important role in the management of one's disease. It especially questions about the role of religious actors in providing the support that patients expect as well as helping them to cope with the disease.
Schreiber J.A., 2011 [90]	Image of God: Effect on coping and psycho‐spiritual outcomes in early breast cancer survivors	To examine the effect of breast cancer survivors’ views of God on religious coping strategies, depression, anxiety, stress, concerns about recurrence, and psychological well‐being.	The belief in an engaged God is significantly related to increased psychological well‐being, decreased psychological distress and decreased concern about recurrence.
Langer Bro et al. 2017 [135]	Kind of blue: A systematic review and meta‐analysis of music interventions in cancer treatment	To analyze and identify the psychological and physical effects of music interventions in cancer treatment.	Music may be a tool in reducing anxiety, mood, and pain among cancer patients in active treatment.
Koerich et al., 2013 [49]	Myocardial revascularization: Strategies for coping with the disease and the surgical process	The study aimed to know the strategies used by patients in coping with coronary heart disease and the surgical process of myocardial revascularization.	The main strategies used by patients to cope with coronary cardiac disease and the process are the presence and support of the family, the quality of interfamily relationships, the use of spiritual resources and participation in rehabilitation programmes that in addition to physical fitness, enable social interactions.
Chatrung et al., 2015 [54]	Wellness and religious coping among Thai individuals living with chronic kidney disease (CKD) in Southern California.	To assist healthcare professionals in developing and implementing educational and supportive interventions for patients with CKD.	The results showed an interrelationships of four (4) emerging categories to have a direct impact on chronic disease‐ family relations, social support, knowledge/awareness and religious coping affect self‐care and how one manages one's life.

## 4 Discussion

This review aimed to identify, compare and synthesise published literature regarding the experiences of adaptation with chronic diseases. The evidence synthesised in this review is significant for two primary reasons. First, through a meticulous and structured approach [35], eight (8) themes related to adaptiveness emerged: maladaptive stress strategies, maintaining ‘normalcy’, medication use, emotional factors, expanding social networks, therapeutic interventions, CTs and the influence of religion/music/nature. The findings presented here illuminate recurring patterns that highlight the complexities of adaptation to chronic disease. These patterns often depend on intricate interactions among the patient, the disease, the caregiving system, the medical care framework, and the broader societal and environmental contexts. The eight (8) overarching themes emphasise the multidimensional nature inherent in managing chronic ailments.

Furthermore, individuals living with chronic conditions face a blend of interconnected factors that affect their ability to engage in self‐management, extending beyond mere symptom control. The thematic insights reveal how these individuals effectively employ problem‐focused adaptation approaches to tackle health challenges while maintaining dignity and independence in pursuit of normalcy [1].

Within this framework, a range of emotions arises regarding their chronic conditions, consistent with previous research [139]. Those dealing with chronic illnesses navigate a complex emotional landscape, which includes negative feelings such as depression, anxiety and sadness, alongside occasional moments of happiness during their adaptation journey. The effort to cultivate a positive outlook through emotional, adaptive approaches such as prayer, listening to music, appreciating nature, distraction and humour further highlights this dynamic [2].

The review also emphasises that adaptation to chronic conditions is not solely an individual endeavour but unfolds within a complex web of environmental influences [140]. The experience of living with chronic conditions intertwines with professional networks and wider social spheres, including family, SO and healthcare providers. Moreover, the literature illuminates the lived experiences associated with chronic conditions, revealing the potential for disruptions in personal biography and shifts in identity. This necessitates acknowledging individuals’ intricate histories, identities and life contexts within the treatment framework.

Furthermore, the review enhances the understanding of the chronic nature of these conditions, spanning their biological foundations to the intimate, subjective experiences of illness. The array of themes encompassing both internal and external strategies underscores the inherently ‘biopsychosocial’ nature of chronic diseases, highlighting that outcomes of similar clinical conditions can vary significantly based on social expectations, available opportunities and resource accessibility (financial, human and infrastructural), ultimately shaping an individual's life trajectory [3].

### 4.1 Strengths and Limitations

This review adhered to the guidelines established by the PRISMA 2020, ensuring a rigorous and systematic approach. A notable strength of this review lies in its comprehensive search strategy, designed to encompass all relevant articles aligned with the review's objectives. However, it is acknowledged that the inclusiveness of the systematic search was inherently limited by the deliberate exclusion of grey literature and non‐English publications. The review successfully included data from a broad cohort representing diverse populations. Still, it is essential to note the predominance of studies from Western regions, with Asia represented to a lesser extent, whereas studies from SSA were noticeably few. This geographical imbalance may limit the generalisability of findings to contexts within SSA, given that cultural differences could impact the conceptualisation and engagement in self‐management of chronic conditions.

Certain inherent limitations exist in this review, particularly the decision not to conduct a meta‐analysis. This choice was driven by the significant heterogeneity observed across the various studies. The research methodologies included in this review ranged from quantitative designs to qualitative frameworks and mixed‐methods approaches. The noticeable divergence in methodologies, variables investigated, data requirements and research objectives rendered a consolidated synthesis impractical. Consequently, the analytical framework adopted here utilised thematic and content analysis, which is appropriate for the diverse methodological landscape. Therefore, due to the apparent dissimilarities among study attributes, a distinct presentation of each study's outcomes was provided, coupled with comparative assessments across common themes to identify overarching patterns or research trajectories.

This review rigorously followed the PRISMA 2020 guidelines and employed the PICO framework to ensure systematic and comprehensive scrutiny. Although the breadth of the search strategy is commendable, there are concerns regarding the non‐inclusion of grey literature and non‐English publications. The review's commendable inclusivity of diverse participant cohorts is acknowledged, but there remains an awareness of the geographical skew. Including diverse methodologies allows for a brief presentation of overall conclusions drawn from the analysis of multiple studies. However, this brevity is a consequence of the differences in study designs, prompting the approach of identifying meaningful patterns across studies by analysing their themes and content, thereby enabling the researchers to find common threads despite their methodological and thematic diversity. Additionally, this review concentrated on articles about the four major global causes of death or disease. Consequently, the 54 reviewed articles do not encompass all known chronic diseases, and studies not published in English were omitted, limiting insights into the experiences of patients with chronic diseases documented in non‐English publications.

Furthermore, most studies reflect the experiences of patients treated in high‐income countries, with only a handful from SSA. Finally, it is essential to highlight that the synthesis process undertaken here is intricately tied to the interpretative lens employed. This may allow for potential misinterpretations of original experiences or analyses. Nevertheless, the application of the PRISMA 2020 synthesis methodology helped mitigate the degree of subjectivity associated with the researchers’ interpretations. Despite these limitations, this review presents a comprehensive fusion of evidence regarding the diverse strategies employed by individuals facing the challenges of chronic diseases. In summary, the review's strengths underscore its methodological rigour and comprehensiveness, whereas the limitations highlight critical considerations for future research and practice in the field. Addressing these challenges with transparency and scholarly rigour enables the review to contribute meaningfully to the existing literature on adaptation among individuals with chronic diseases.

## 5 Conclusion

This SR identified eight thematic categories of adaptive coping strategies employed by individuals living with chronic illnesses. These span internal strategies such as maintaining normalcy, medical and behavioural management, emotional and psychological factors and stress‐coping approaches, alongside external strategies, including social support, CTs, therapeutic interventions and religious or spiritual practices. Together, these strategies highlight the multidimensional nature of coping, integrating physical, psychological, social and spiritual resources.

The synthesis of evidence across cancer, COPD, diabetes and heart disease demonstrates that person‐centred, culturally sensitive and context‐specific interventions can support adaptive coping and improve QoL. In addition, this review contributes to existing knowledge by providing an integrated, cross‐condition perspective that draws together adaptive strategies often examined in isolation. This consolidated understanding offers a framework for healthcare providers, policymakers and researchers to design more holistic and contextually relevant interventions. Future research should examine the interplay between these strategies and health outcomes, with particular attention to resource‐limited settings and underrepresented populations.

## Author Contributions

**Andrew Kweku Conduah**: conceptualisation, methodology, data curation, formal analysis, writing – original draft, writing – review and editing, supervision. **Mary Naana Essiaw**: validation, writing – review and editing.  **Sebastian Hadjor Ofoe**: data curation, writing – review and editing, project administration.

## Ethics Statement

The authors have nothing to report.

## Consent

The authors have nothing to report.

## Conflicts of Interest

The authors declare no conflicts of interest.

## Reporting Guidelines

Fig share: Conduah, Andrew (2023). A Systematic Review of Coping Strategies among Patients with Cancer, Chronic Obstructive Pulmonary Disease, Diabetes Mellitus and Coronary Heart Diseases figshare. Dataset. https://doi.org/10.6084/m9.figshare.22892123.v1

Data are available under the terms of the Creative Commons Attribution 4.0 International license (CC‐BY 4.0).

## Data Availability

All data relevant to the study are included in the manuscript and additional supporting files.
